# Supplementary material for: Sirtuin 6 inhibition protects against glucocorticoid-induced skeletal muscle atrophy by regulating IGF/PI3K/AKT signaling
Source: Nat Commun. 2022 Sep 15;13:5415. doi: 10.1038/s41467-022-32905-w (PMC9478160; doi:10.1038/s41467-022-32905-w)
Supplement: Supplementary file 1 — Supplementary Information [file 41467_2022_32905_MOESM1_ESM.pdf]

Sirtuin 6 inhibition protects against glucocorticoid-induced skeletal muscle atrophy by regulating IGF/PI3K/AKT signaling

Sneha Mishra[1,$], Claudia Cosentino[2,$], Ankit Kumar Tamta[1], Danish Khan[1], Shalini Srinivasan[1], Venkatraman Ravi[1], Elena Abbotto[3], Bangalore Prabhashankar Arathi[1], Shweta Kumar[1], Aditi Jain[4], Anand S. Ramaian[5], Shruti M. Kizkekra[1], Raksha Rajagopal[1], Swathi Rao[1], Swati Krishna[1], Ninitha Asirvatham-Jeyaraj[6], Elizabeth R. Haggerty[2], Dafne M. Silberman[7], Irwin J. Kurland[7], Ravindra P. Veeranna[8], Tamilselvan Jayavelu[5], Santina Bruzzone[3], Raul Mostoslavsky[2,#], Nagalingam R. Sundaresan[1,#]

[1] Department of Microbiology and Cell Biology, Indian Institute of Science, Bengaluru, India.

[2] The Massachusetts General Hospital Cancer Center, Harvard Medical School, Boston, MA, USA.

[3] Department of Experimental Medicine, Section of Biochemistry, and CEBR, University of Genoa, Genoa, Italy.

[4] Centre for BioSystems Science and Engineering, Indian Institute of Science, Bengaluru, India.

[5] Department of Biotechnology, Anna University, Chennai, India.

[6] Department of Biotechnology, Indian Institute of Technology, Chennai, India.

[7] Centro de Estudios Farmacologicos y Botanicos (CEFYBO-CONICET), Catedra de Farmacologia, Facultad de Medicina, UBA, Buenos Aires, Argentina

[8] Albert Einstein College of Medicine, Bronx, NY, USA

[9] Department of Biochemistry, CSIR- Central Food Technological Research Institute, Mysuru, India.

[$] Equal contribution.

[#] Correspondence should be addressed to: rmostoslavsky@mgh.harvard.edu, rsundaresan@iisc.ac.in

Supplementary Figures and Figure Legends

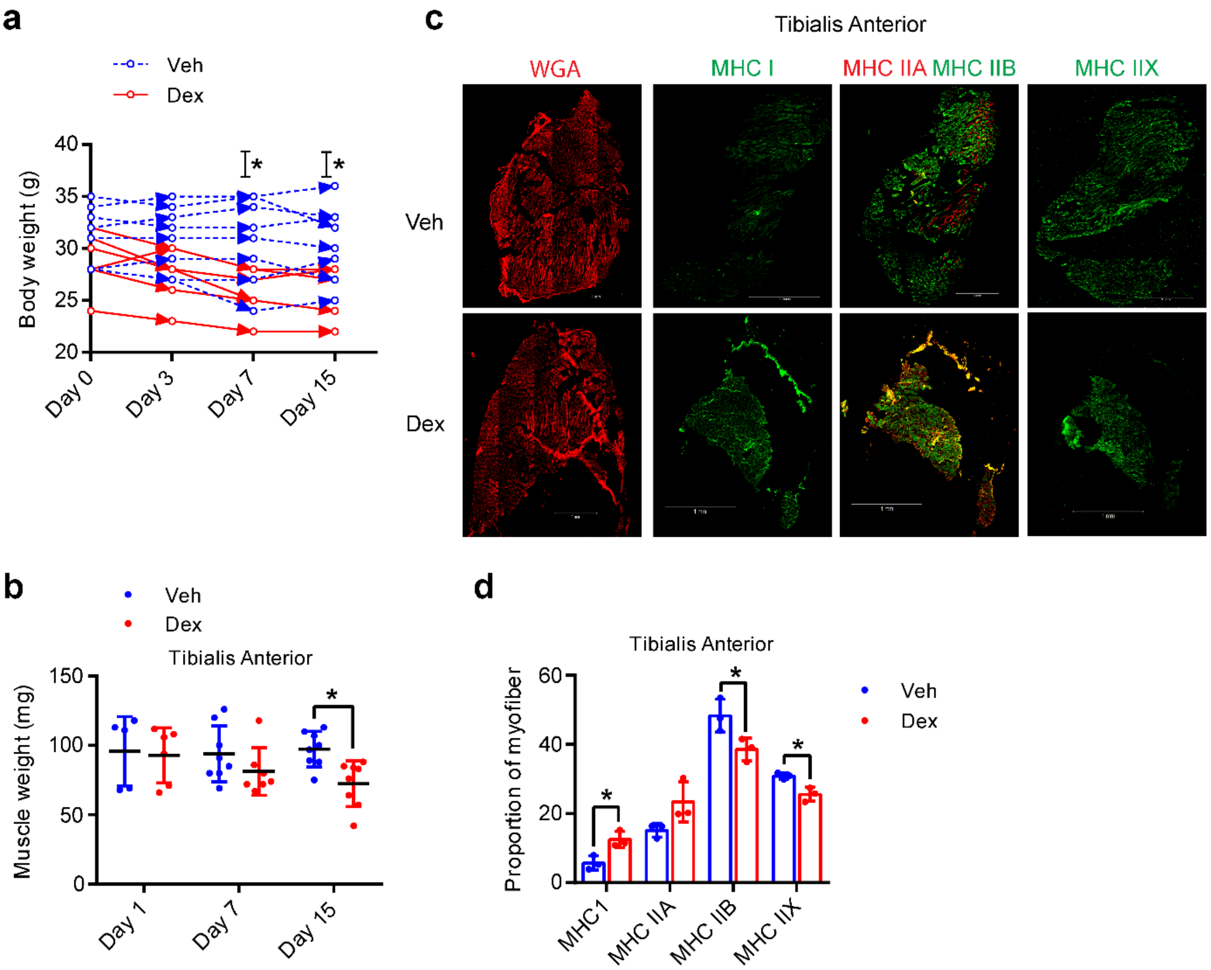

Supplementary Figure 1: Characterization of dexamethasone treated mice tibialis anterior muscle

a Change in body weight with time for each mouse after Dex (10mg/kg/day) administration. n = 8. **b** Time dependent loss in TA absolute muscle mass from Dex (10mg/kg/day) administered mice. Day 1-Veh n = 5, Day 1-Dex n= 6, Day 7-Veh n= 8, Day 7-Dex n= 7, Day 15-Veh n=8, Day 15-Dex n= 8. **c** Representative images showing wheat germ agglutinin (WGA; red) to determine CSA and MHC I (green), MHC IIA MHC IIB (MHC IIA; red, MHC IIB; green), MHC IIX (green) staining to determine proportion of muscle fiber types in Dex (10mg/kg/day) administered TA muscle. Scale bar= 1 mm. n = 3. **d** Quantification for proportion of myofiber for mice described in **c**. n = 3. Data presented as mean ± s.d, *p < 0.05. Two-way repeated measure ANOVA with Bonferroni correction was used for statistical analysis **a**. Two-way ANOVA with Bonferroni post hoc test was used for statistical analysis **b**. Two-tailed unpaired Student's t-test was used for statistical analysis **d**. Source data are provided as a Source Data file.

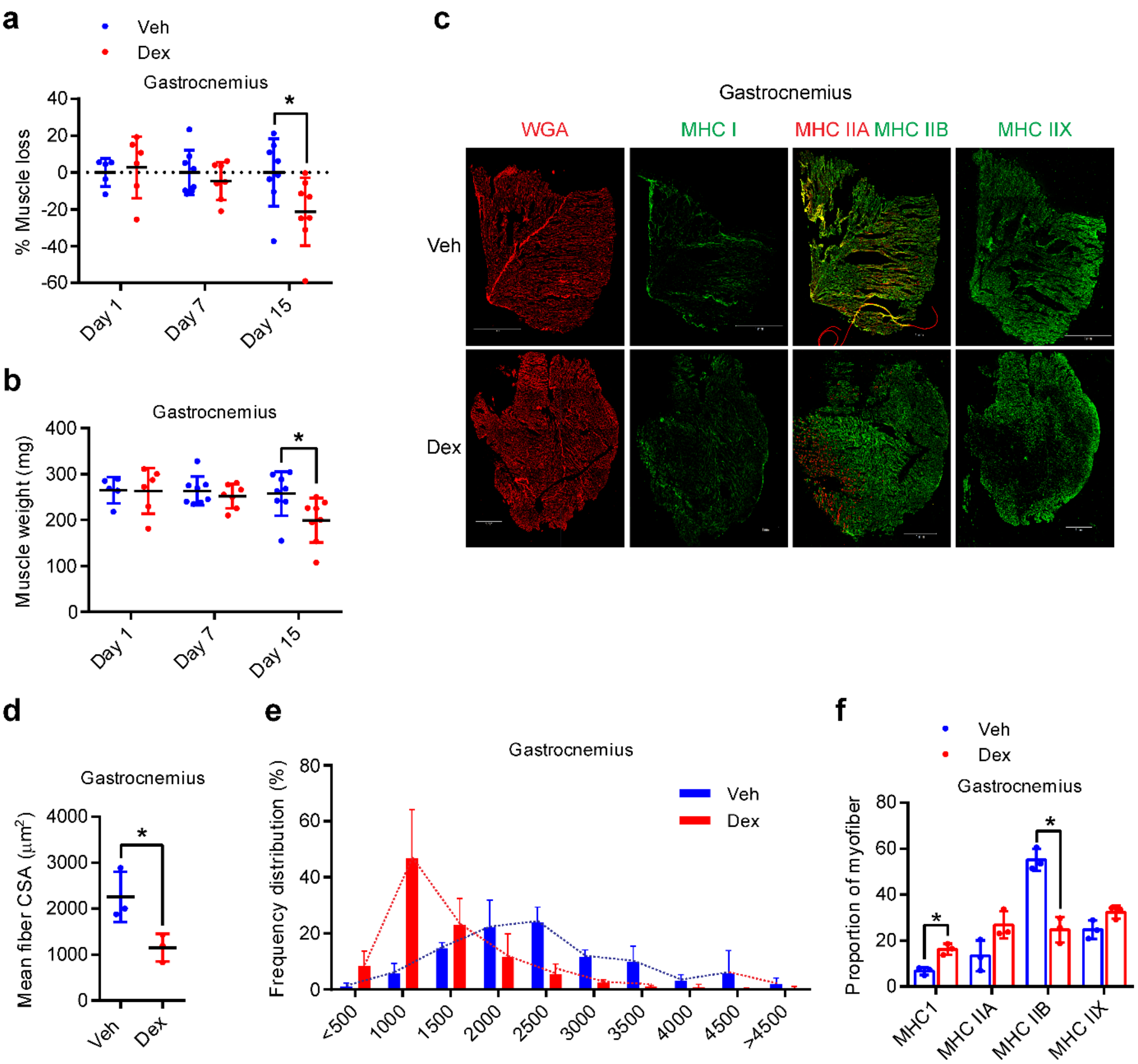

Supplementary Figure 2: Characterization of dexamethasone treated mice gastrocnemius muscle

a Time dependent loss in gastrocnemius muscle mass from Dex (10mg/kg/day) administered mice. % of muscle loss in Dex group is shown relative to Veh injected mice from each time-point. Day 1-Veh n = 5, Day 1-Dex n= 6, Day 7-Veh n= 8, Day 7-Dex n= 7, Day 15-Veh n=8, Day 15-Dex n= 8. **b** Time dependent absolute loss in gastrocnemius muscle mass from Dex (10mg/kg/day) administered mice. Day 1-Veh n = 5, Day 1-Dex n= 6, Day 7-Veh n= 8, Day 7-Dex n= 7, Day 15-Veh n=8, Day 15-Dex n= 8. **c** Representative images showing WGA (red) to determine CSA and MHC I (green), MHC IIA MHC IIB (MHC IIA; red, MHC IIB; green), MHC IIX (green) staining to determine proportion of muscle fiber types in Dex (10mg/kg/day) administered gastrocnemius muscle. Scale bar= 1 mm. n = 3. **d** Mean fiber CSA in mice described in **c**. n = 3. **e** Frequency distribution of fiber CSA in mice described in **c.** n = 3. **f** Quantification for proportion of MHC I, MHC IIA, MHC IIB, and MHC IIX myofiber in mice

described in **c**. n = 3. Data presented as mean ± s.d, *p < 0.05. Two-way ANOVA with Bonferroni post hoc test was used for statistical analysis **a, b**. Two-tailed unpaired Student's t-test was used for statistical analysis **d, f**. Source data are provided as a Source Data file.

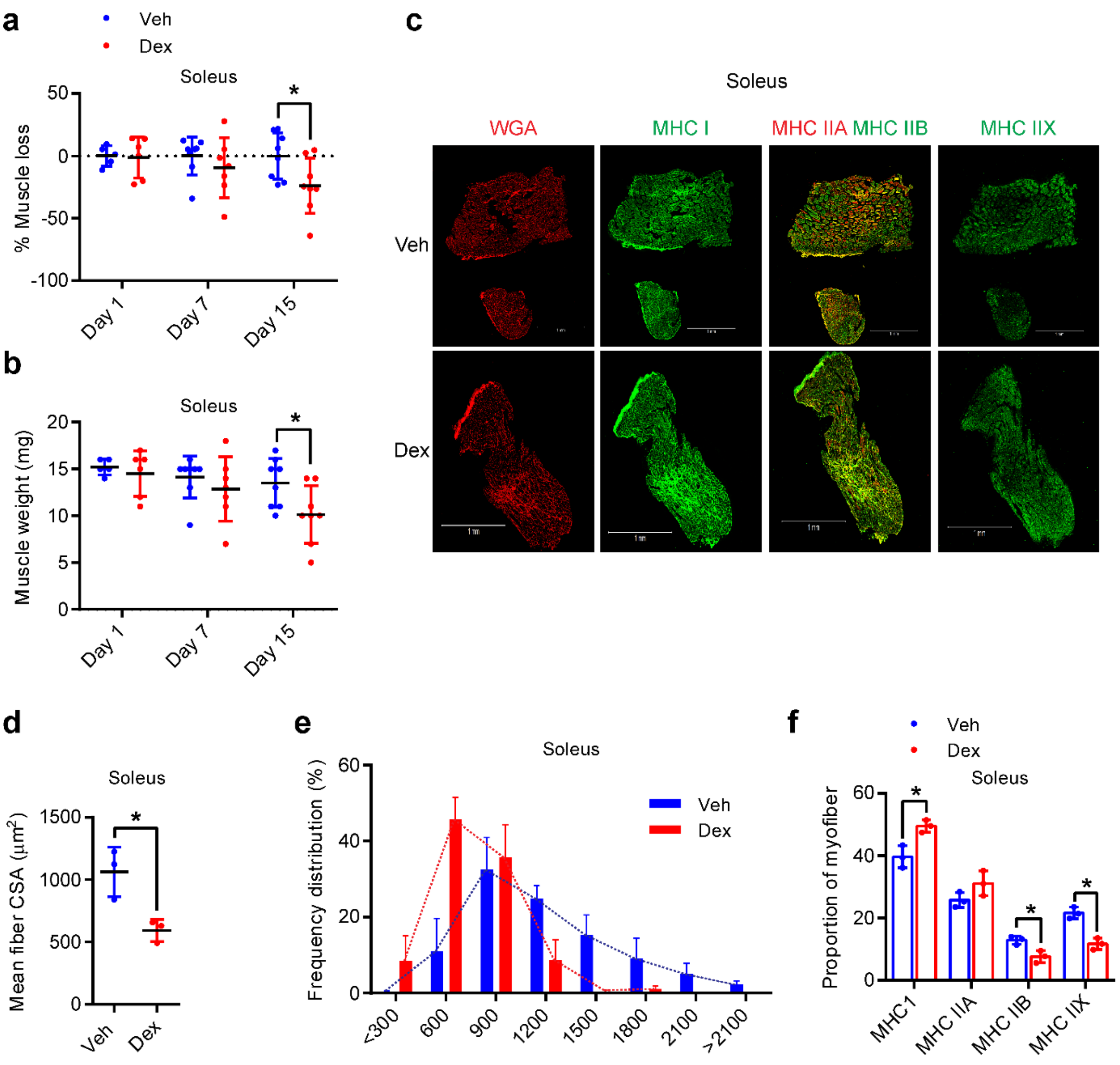

Supplementary Figure 3: Characterization of dexamethasone treated mice soleus muscle

a Time dependent percentage (%) loss in soleus muscle mass from Dex (10mg/kg/day) administered mice. % of muscle loss in Dex group is shown relative to Veh injected mice from each time-point. Day 1-Veh n = 5, Day 1-Dex n= 6, Day 7-Veh n= 8, Day 7-Dex n= 7, Day 15-Veh n=8, Day 15-Dex n= 8. **b** Time dependent absolute loss in soleus muscle mass from Dex (10mg/kg/day) administered mice. Day 1-Veh n = 5, Day 1-Dex n= 6, Day 7-Veh n= 8, Day 7-Dex n= 7, Day 15-Veh n=8, Day 15-Dex n= 8. **c** Representative images showing WGA (red) to determine CSA and MHC I (green), MHC IIA MHC IIB (MHC IIA; red, MHC IIB; green), MHC IIX (green) staining to determine proportion of muscle fiber types in Dex (10mg/kg/day) administered soleus muscle. Scale bar= 1 mm. n = 3. **d** Mean fiber CSA in Dex (10mg/kg/day) administered mice described in **c**. n = 3. **e** Frequency distribution of fiber CSA in Dex (10mg/kg/day) administered mice described in **c**. n = 3. **f** Quantification for proportion of MHC I, MHC IIA, MHC IIB, and MHC IIX myofiber described in **c**. n = 3. Data presented as mean ± s.d, *$p < 0.05$. Two-way ANOVA with Bonferroni post hoc test was used for statistical analysis **a, b**. Two-tailed unpaired Student's t-test was used for statistical analysis **d, f**. Source data are provided as a Source Data file.

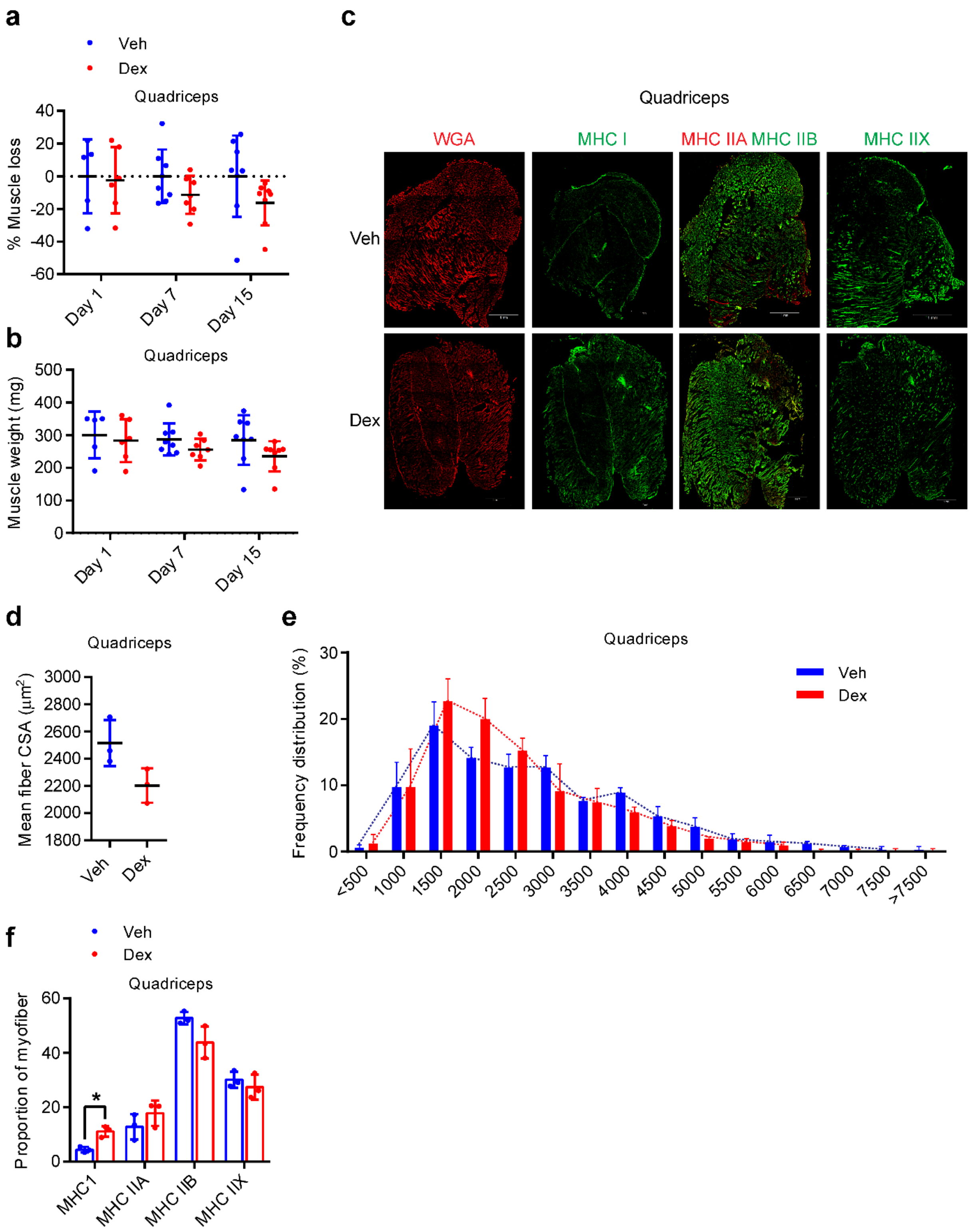

Supplementary Figure 4: Characterization of dexamethasone treated mice quadriceps muscle

a Time dependent percentage (%) loss in quadriceps muscle mass from Dex (10mg/kg/day) administered mice. % of muscle loss in Dex group is shown relative to Veh injected mice from each time-point. Day 1-Veh n = 5, Day 1-Dex n= 6, Day 7-Veh n= 8, Day 7-Dex n= 7, Day 15-

Veh n=8, Day 15-Dex n= 8. **b** Time dependent absolute loss in quadriceps muscle mass from Dex (10mg/kg/day) administered mice. Day 1-Veh n = 5, Day 1-Dex n= 6, Day 7-Veh n= 8, Day 7-Dex n= 7, Day 15-Veh n=8, Day 15-Dex n= 8. **c** Representative images showing WGA (red) to determine CSA and MHC I (green), MHC IIA MHC IIB (MHC IIA; red, MHC IIB; green), MHC IIX (green) staining to determine proportion of muscle fiber types in Dex (10mg/kg/day) administered quadriceps muscle. Scale bar= 1 mm. n = 3. **d** Scatterplot showing mean fiber CSA in mice described in **c**. n = 3. **e** Frequency distribution of fiber CSA in mice described in **c**. n = 3. **f** Quantification for proportion of MHC I, MHC IIA, MHC IIB, and MHC IIX myofiber in mice described in **c**. n = 3. Data presented as mean ± s.d, *p < 0.05. Two-way ANOVA with Bonferroni post hoc test was used for statistical analysis **a, b**. Two-tailed unpaired Student's t-test was used for statistical analysis **d, f**. Source data are provided as a Source Data file.

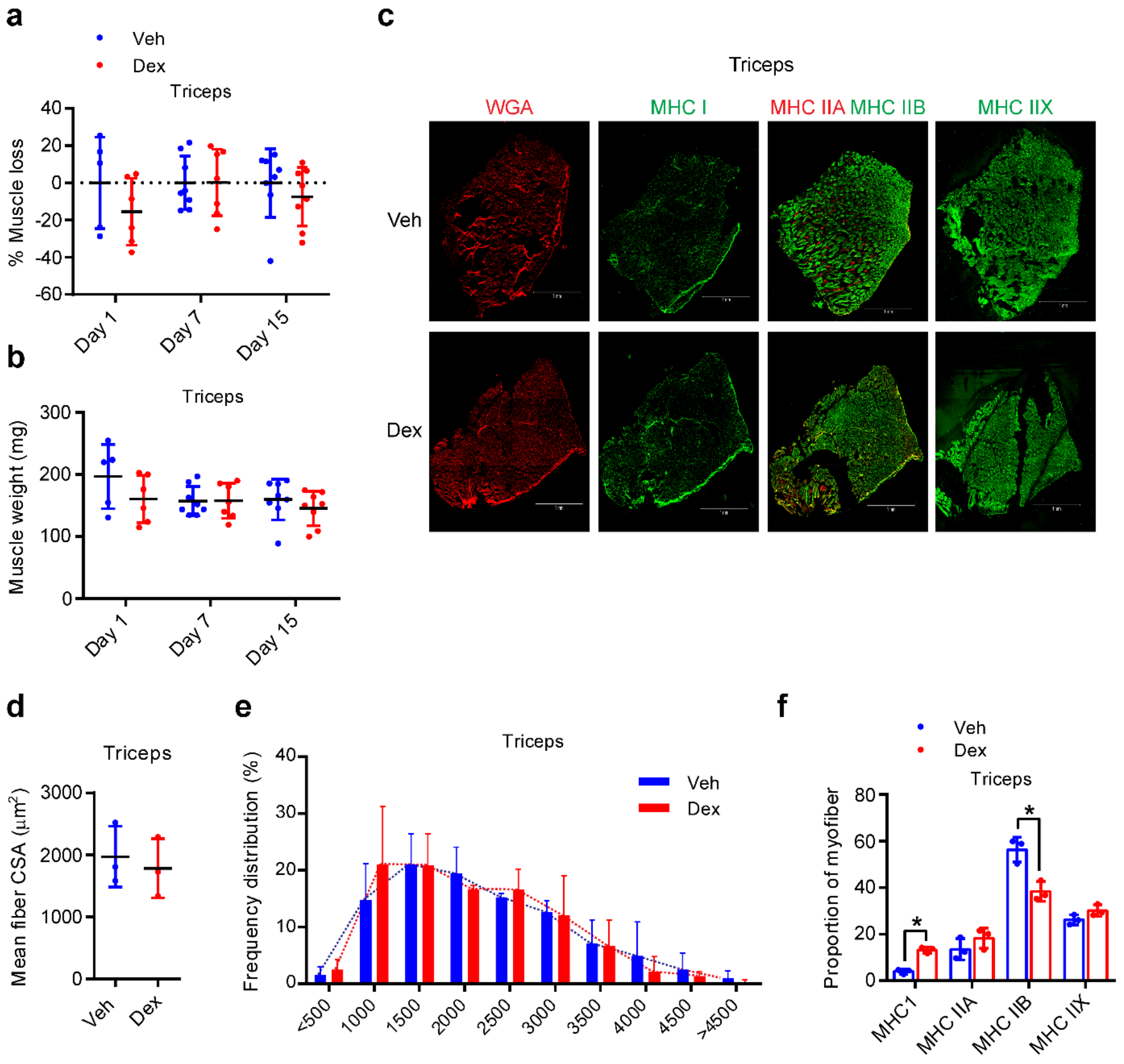

Supplementary Figure 5: Characterization of dexamethasone treated mice triceps muscle

a Time dependent percentage (%) loss in triceps muscle mass from Dex (10mg/kg/day) administered mice. % of muscle loss in Dex group is shown relative to Veh injected mice from each time-point. Day 1-Veh n = 5, Day 1-Dex n= 6, Day 7-Veh n= 8, Day 7-Dex n= 7, Day 15-Veh n=8, Day 15-Dex n= 8. **b** Time dependent absolute loss in triceps muscle mass from Dex (10mg/kg/day) administered mice. Day 1-Veh n = 5, Day 1-Dex n= 6, Day 7-Veh n= 8, Day 7-Dex n= 7, Day 15-Veh n=8, Day 15-Dex n= 8. **c** Representative images showing WGA (red) to determine CSA and MHC I (green), MHC IIA MHC IIB (MHC IIA; red, MHC IIB; green), MHC IIX (green) staining to determine proportion of muscle fiber types in Dex (10mg/kg/day) administered triceps muscle. Scale bar= 1 mm. n = 3. **d** Scatterplot showing mean fiber CSA in mice described in **c**. n = 3. **e** Frequency distribution of fiber CSA in mice described in **c**. n = 3. **f** Quantification for proportion of MHC I, MHC IIA, MHC IIB, and MHC IIX myofiber in mice described in **c**. n = 3. Data presented as mean ± s.d, *p < 0.05. Two-way ANOVA with Bonferroni post hoc test was used for statistical analysis **a, b**. Two-tailed unpaired Student's t-test was used for statistical analysis **d, f**. Source data are provided as a Source Data file.

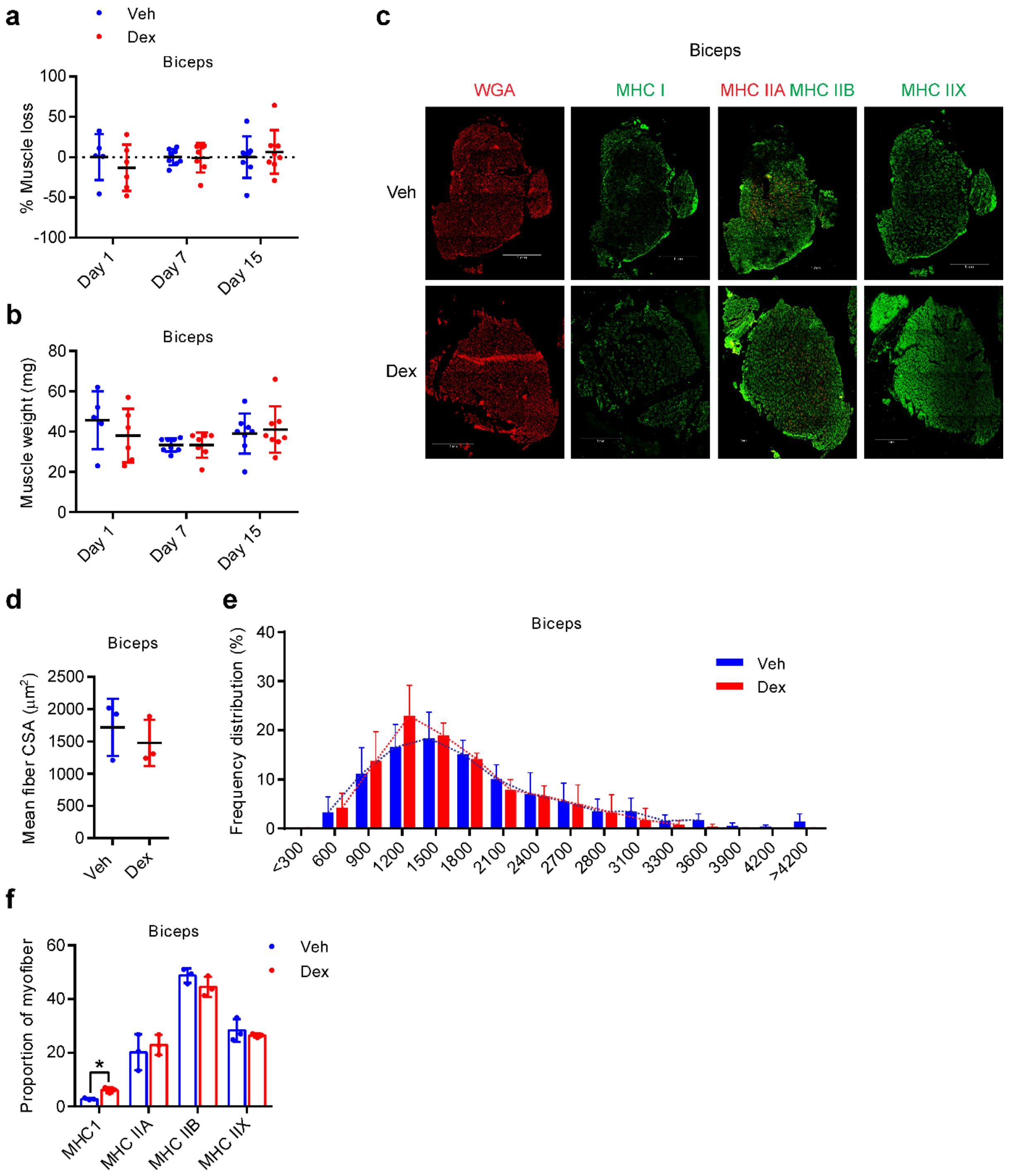

Supplementary Figure 6: Characterization of dexamethasone treated mice biceps muscle

a Time dependent percentage (%) loss in biceps muscle mass from Dex (10mg/kg/day) administered mice. % of muscle loss in Dex group is shown relative to Veh injected mice from each time-point. Day 1-Veh n = 5, Day 1-Dex n= 6, Day 7-Veh n= 8, Day 7-Dex n= 7, Day 15-Veh n=8, Day 15-Dex n= 8. **b** Time dependent absolute loss in biceps muscle mass from Dex (10mg/kg/day) administered mice. Day 1-Veh n = 5, Day 1-Dex n= 6, Day 7-Veh n= 8, Day 7-Dex n= 7, Day 15-Veh n=8, Day 15-Dex n= 8. **c** Representative images showing WGA (red)

to determine CSA and MHC I (green), MHC IIA MHC IIB (MHC IIA; red, MHC IIB; green), MHC IIX (green) staining to determine proportion of muscle fiber types in Dex (10mg/kg/day) administered biceps muscle. Scale bar= 1 mm. n = 3. **d** Scatterplot showing mean fiber CSA in mice described in **c**. n = 3. **e** Frequency distribution of fiber CSA in mice described in **c**. n = 3. **f** Quantification for proportion of MHC I, MHC IIA, MHC IIB, and MHC IIX myofiber in mice described in **c**. n = 3. Data presented as mean ± s.d, *$p < 0.05$. Two-way ANOVA with Bonferroni post hoc test was used for statistical analysis **a, b**. Two-tailed unpaired Student's t-test was used for statistical analysis **d, f**. Source data are provided as a Source Data file.

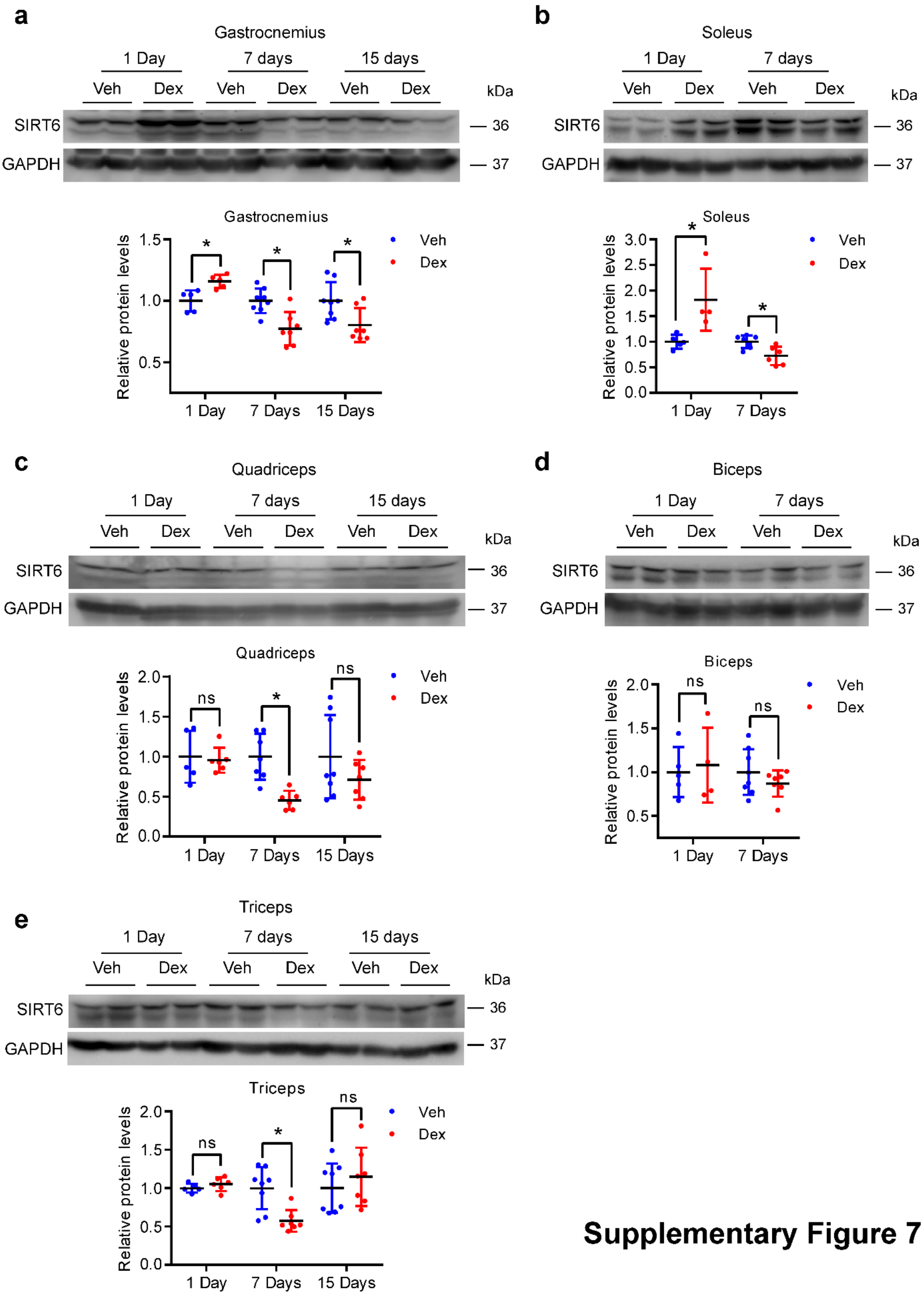

Supplementary Figure 7: Dexamethasone increases SIRT6 levels in gastrocnemius and soleus muscles

a Western blot (top) and relative quantification (bottom) of SIRT6 protein levels in gastrocnemius muscle of mice injected with Dex (10mg/kg/day). Day 1-Veh n = 5, Day 1-Dex n= 5, Day 7-Veh n= 8, Day 7-Dex n= 7, Day 15-Veh n=8, Day 15-Dex n= 7. **b** Western blot (top) and relative quantification (bottom) of SIRT6 protein levels in soleus muscle of mice injected with Dex (10mg/kg/day). Day 1-Veh n = 5, Day 1-Dex n= 4, Day 7-Veh n= 7, Day 7-Dex n= 6. **c** Western blot (top) and relative quantification (bottom) of SIRT6 protein levels in quadriceps muscle of mice injected with Dex (10mg/kg/day). Day 1-Veh n = 5, Day 1-Dex n= 6, Day 7-Veh n= 7, Day 7-Dex n= 6, Day 15-Veh n=8, Day 15-Dex n= 7. **d** Western blot (top) and relative quantification (bottom) of SIRT6 protein levels in biceps muscle of mice injected with Dex (10mg/kg/day). Day 1-Veh n = 5, Day 1-Dex n= 4, Day 7-Veh n= 8, Day 7-Dex n= 7. **e** Western blot (top) and relative quantification (bottom) of SIRT6 protein levels in triceps muscle of mice injected with Dex (10mg/kg/day). Day 1-Veh n = 5, Day 1-Dex n= 6, Day 7-Veh n= 8, Day 7-Dex n= 7, Day 15-Veh n=8, Day 15-Dex n= 7. Data presented as mean ± s.d, *p < 0.05. Two-tailed unpaired Student's t-test was used for statistical analysis **a-e**. Source data are provided as a Source Data file.

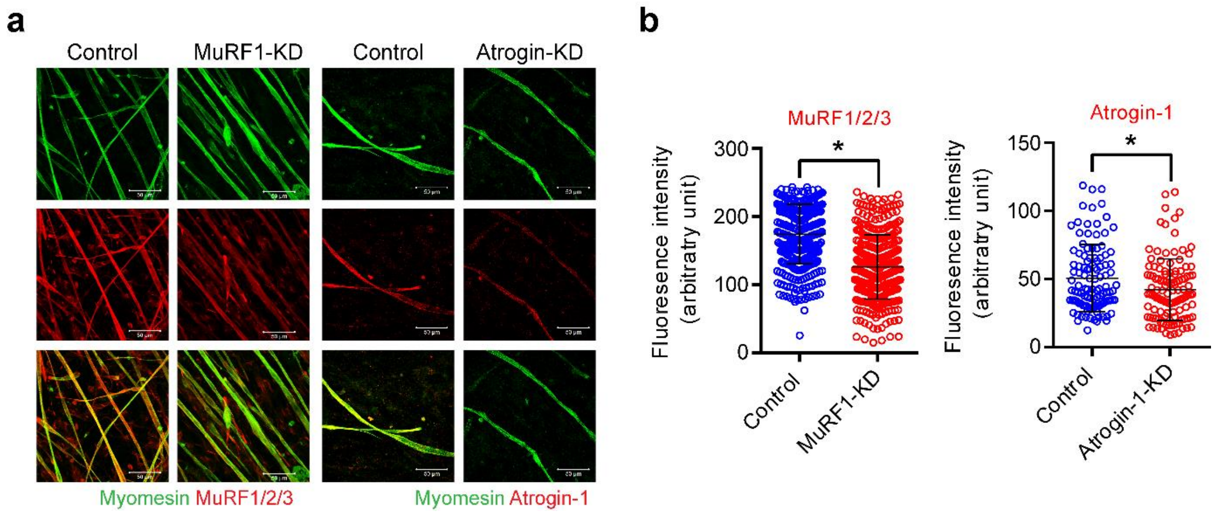

Supplementary Figure 8: Validation of siRNA mediated depletion of MuRF-1 and Atrogin-1

a Representative confocal image of MuRF-1/2/3 or Atrogin-1 levels to confirm RNAi-mediated depletion of MuRF-1 (left) or Atrogin-1 (right) in primary myotube transfected with control siRNA or siRNA targeting MuRF-1 or Atrogin-1. MuRF-1/2/3 or Atrogin-1 were stained in red and myomesin in green. Scale bar = 50 µm. **b** Scatterplot showing MuRF-1/2/3 (left) or Atrogin-1 (right) fluorescence intensity in myotubes described in **a**. MuRF-1/2/3 (Control n = 407, MuRF-1-KD n = 377), Atrogin-1 (Control n = 110, Atrogin-1-KD n = 119). Data presented as mean ± s.d, *p < 0.05. Two-tailed unpaired Student's t-test was used for statistical analysis **b**. Source data are provided as a Source Data file.

a

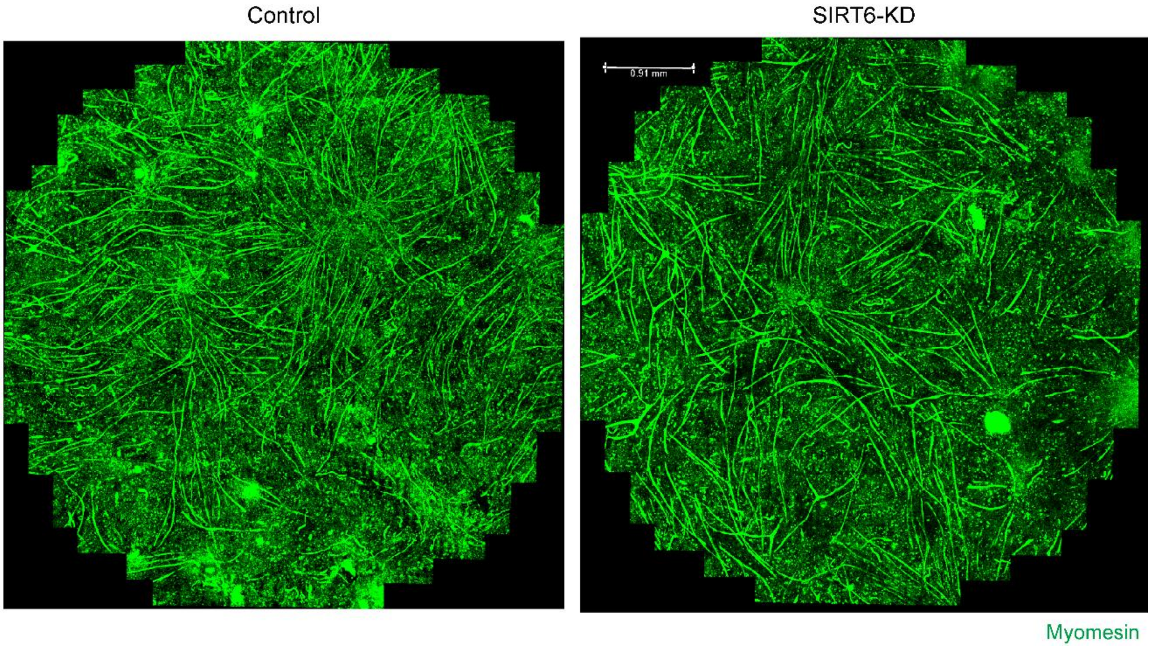

Supplementary Figure 9: SIRT6 depletion increases myotube diameter

a Tile scan images of quarter of a cover slip of control and SIRT6 depleted (SIRT6-KD) myotubes acquired using automated confocal microscopy.

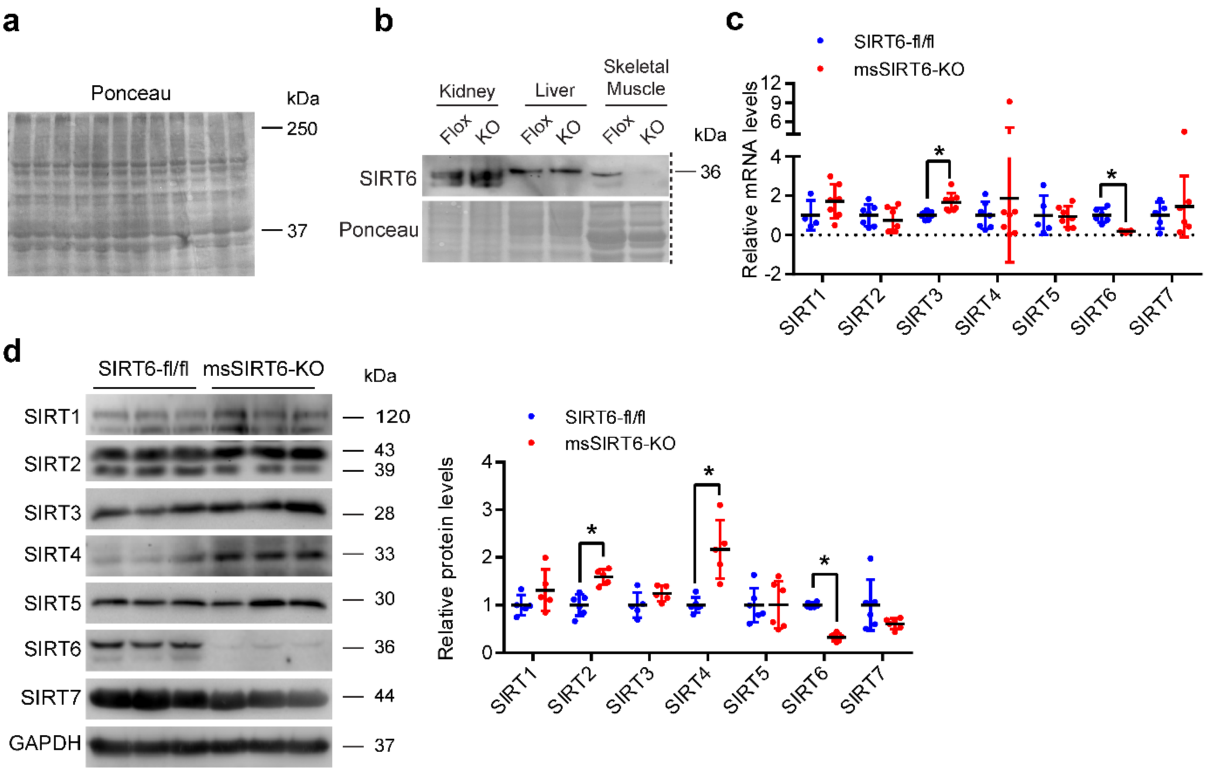

Supplementary Figure 10: Screening of Sirtuins level in muscle-specific SIRT6 deficient mice

a Ponceau stained western blot showing protein equal loading for Figure 4A (right). **b** Western blotting showing specific depletion of SIRT6 in skeletal muscle but not in other tissues tested in muscle-specific SIRT6 knockout (msSIRT6-KO) mice. **c** qPCR analysis of relative mRNA levels of all Sirtuin isoforms in SIRT6-fl/fl and msSIRT6-KO mice gastrocnemius muscle. SIRT1 (SIRT6-fl/fl n = 4, msSIRT6-KO n = 7), SIRT2 (SIRT6-fl/fl and msSIRT6-KO n = 7), SIRT3 (SIRT6-fl/fl n = 7, msSIRT6-KO n = 8), SIRT4 (SIRT6-fl/fl and msSIRT6-KO n = 7), SIRT5 (SIRT6-fl/fl n = 5, msSIRT6-KO n = 7), SIRT6 (SIRT6-fl/fl n = 6, msSIRT6-KO n = 5), SIRT7 (SIRT6-fl/fl n = 5, msSIRT6-KO n = 6). **d** Western blot (left) and relative quantification (right) of protein levels of all Sirtuin isoforms in SIRT6-fl/fl and msSIRT6-KO mice gastrocnemius muscle. SIRT1 (SIRT6-fl/fl and msSIRT6-KO n = 5), SIRT2 (SIRT6-fl/fl n = 6, msSIRT6-KO n = 5), SIRT3 (SIRT6-fl/fl and msSIRT6-KO n = 5), SIRT4 (SIRT6-fl/fl n = 6, msSIRT6-KO n = 5), SIRT5 (SIRT6-fl/fl and msSIRT6-KO n = 6), SIRT6 (SIRT6-fl/fl and msSIRT6-KO n = 6), SIRT7 (SIRT6-fl/fl and msSIRT6-KO n = 6). Data presented as mean ± s.d, *$p < 0.05$. Two-tailed unpaired Student's t-test was used for statistical analysis **c, d**. Source data are provided as a Source Data file.

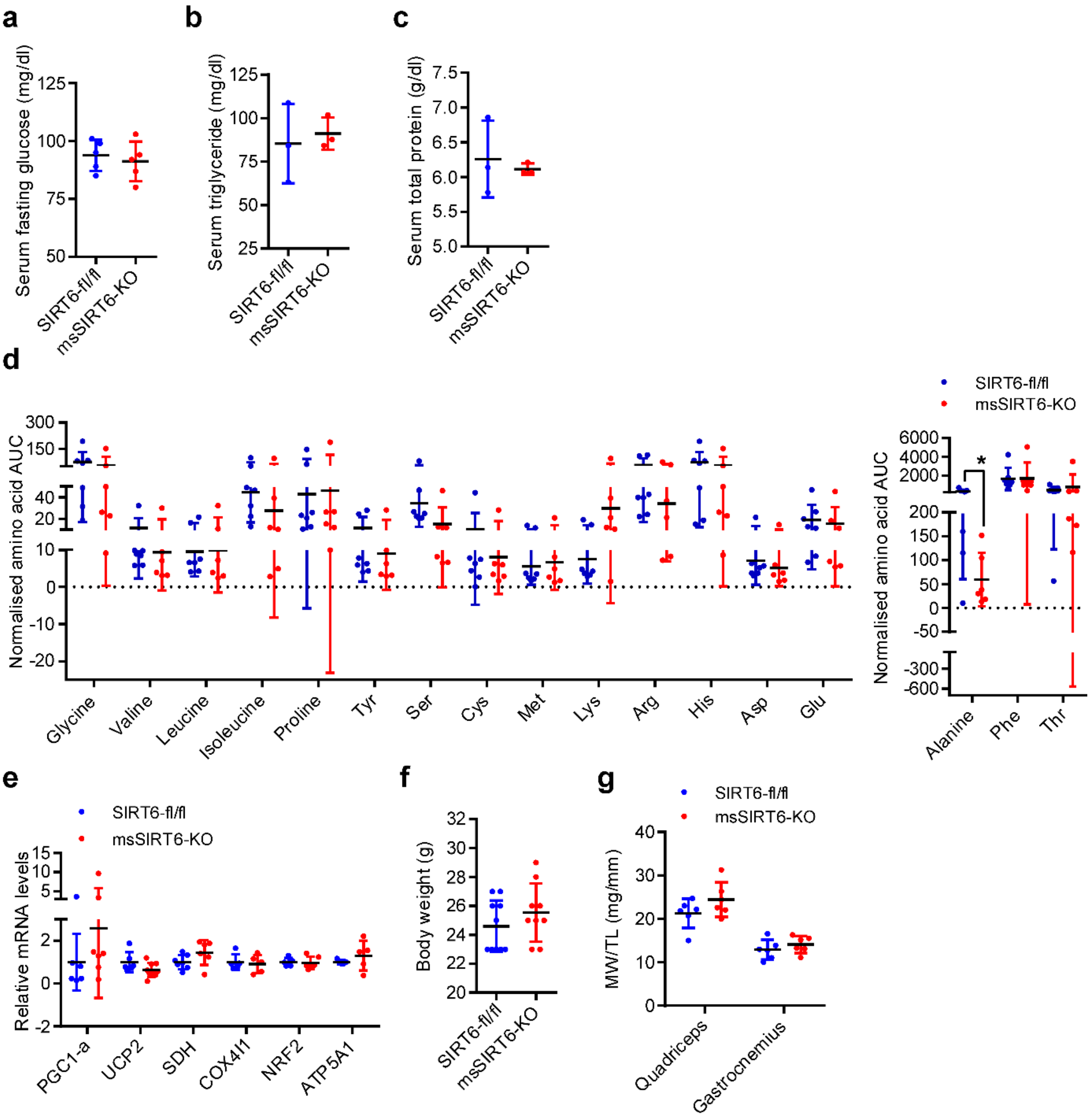

Supplementary Figure 11: Characterization of muscle-specific SIRT6 deficient mice

a Serum fasting glucose levels in SIRT6-fl/fl and msSIRT6-KO mice. n = 5. **b** Serum triglyceride levels in SIRT6-fl/fl and msSIRT6-KO mice. n = 3. **c** Serum total protein levels in SIRT6-fl/fl and msSIRT6-KO mice. n = 3. **d** Normalized amino acids levels in SIRT6-fl/fl and msSIRT6-KO mice muscles measured by HPLC analysis, amino acids with smaller SD (left) and amino acids with high SD (right). SIRT6-fl/fl n = 7, msSIRT6-KO n =6. **e** qPCR analysis for relative expression of mitochondrial genes in gastrocnemius muscle of SIRT6-fl/fl and msSIRT6-KO mice. PGC1-a (SIRT6-fl/fl n = 6, msSIRT6-KO n =7), UCP2 (SIRT6-fl/fl n = 6, msSIRT6-KO n =9), SDH (SIRT6-fl/fl n = 7, msSIRT6-KO n =6), COX4I1 (SIRT6-fl/fl n = 5, msSIRT6-KO n =5), NRF2 (SIRT6-fl/fl n = 5, msSIRT6-KO n =6), ATP5A1 (SIRT6-fl/fl n = 5, msSIRT6-KO n =5). **f** Body weight of SIRT6-fl/fl and msSIRT6-KO mice at 4 months of age.

SIRT6-fl/fl n = 10, msSIRT6-KO n =9. **g** Muscle weight/tibia length (MW/TL) ratio of SIRT6-fl/fl and msSIRT6-KO mice at 4 months of age. n = 6. Data presented as mean ± s.d, $*p < 0.05$. Two-tailed unpaired Student’s t-test was used for statistical analysis **a-g**. Source data are provided as a Source Data file.

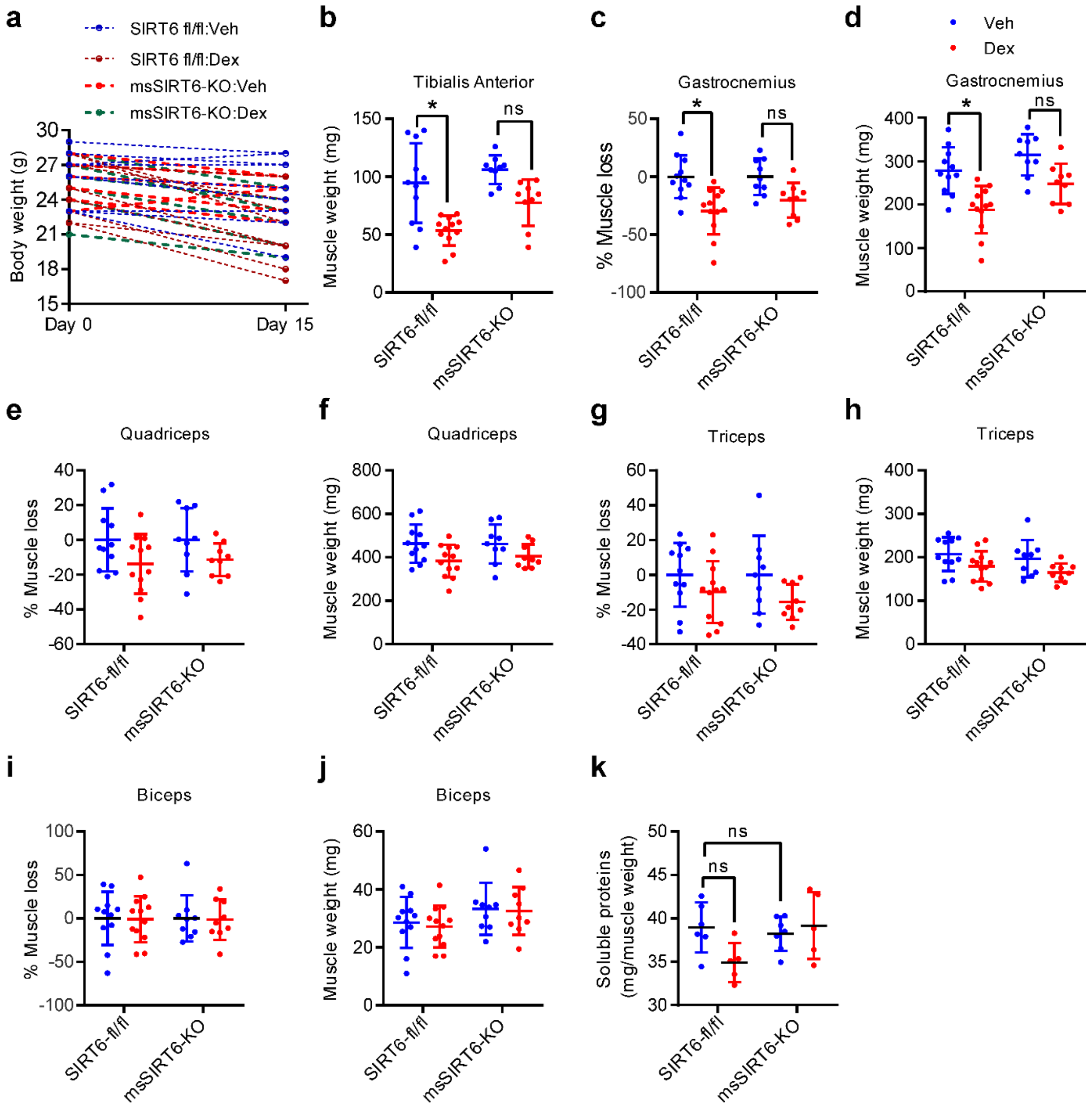

Supplementary Figure 12: Muscle-specific SIRT6 deficient mice are resistant to dexamethasone induced muscle loss

a Change in body weight with time for each SIRT6-fl/fl and msSIRT6-KO mouse after Dex (10mg/kg/day) injection. **b** Loss in absolute tibialis anterior muscle mass in SIRT6-fl/fl and msSIRT6-KO mice described in **a**. **c** Percentage (%) loss in gastrocnemius muscle mass in SIRT6-fl/fl and msSIRT6-KO mice described in **a**. **d** Loss in gastrocnemius absolute muscle mass in SIRT6-fl/fl and msSIRT6-KO mice described in **a**. **e** Percentage (%) loss in quadriceps muscle mass in SIRT6-fl/fl and msSIRT6-KO mice described in **a**. **f** Loss in quadriceps absolute muscle mass in SIRT6-fl/fl and msSIRT6-KO mice described in **a**. **g** Percentage (%) loss in triceps muscle mass in SIRT6-fl/fl and msSIRT6-KO mice described in **a**. **h** Loss in triceps absolute muscle mass in SIRT6-fl/fl and msSIRT6-KO mice described in **a**. **i**

Percentage (%) loss in biceps muscle mass in SIRT6-fl/fl and msSIRT6-KO mice described in **a**. **j** Loss in biceps absolute muscle mass in SIRT6-fl/fl and msSIRT6-KO mice described in **a**. **a-j** SIRT6-fl/fl-Veh n = 11, SIRT6-fl/fl-Dex n = 12, msSIRT6-KO-Veh n = 9, msSIRT6-KO-Dex n = 9. % of muscle loss in SIRT6-fl/fl-Dex or msSIRT6-KO-Dex group is shown relative to Veh injected mice from each genotype. **k** Soluble protein concentration in SIRT6-fl/fl and msSIRT6-KO mice muscle from intraperitoneal Dex (10mg/kg/day) administered mice. SIRT6-fl/fl-Veh n = 6, SIRT6-fl/fl-Dex n = 5, msSIRT6-KO-Veh n = 7, msSIRT6-KO-Dex n = 5. Data presented as mean ± s.d, *p < 0.05, ns - not significant. Two-way ANOVA with Bonferroni post hoc test was used for statistical analysis **b-k**. Source data are provided as a Source Data file.

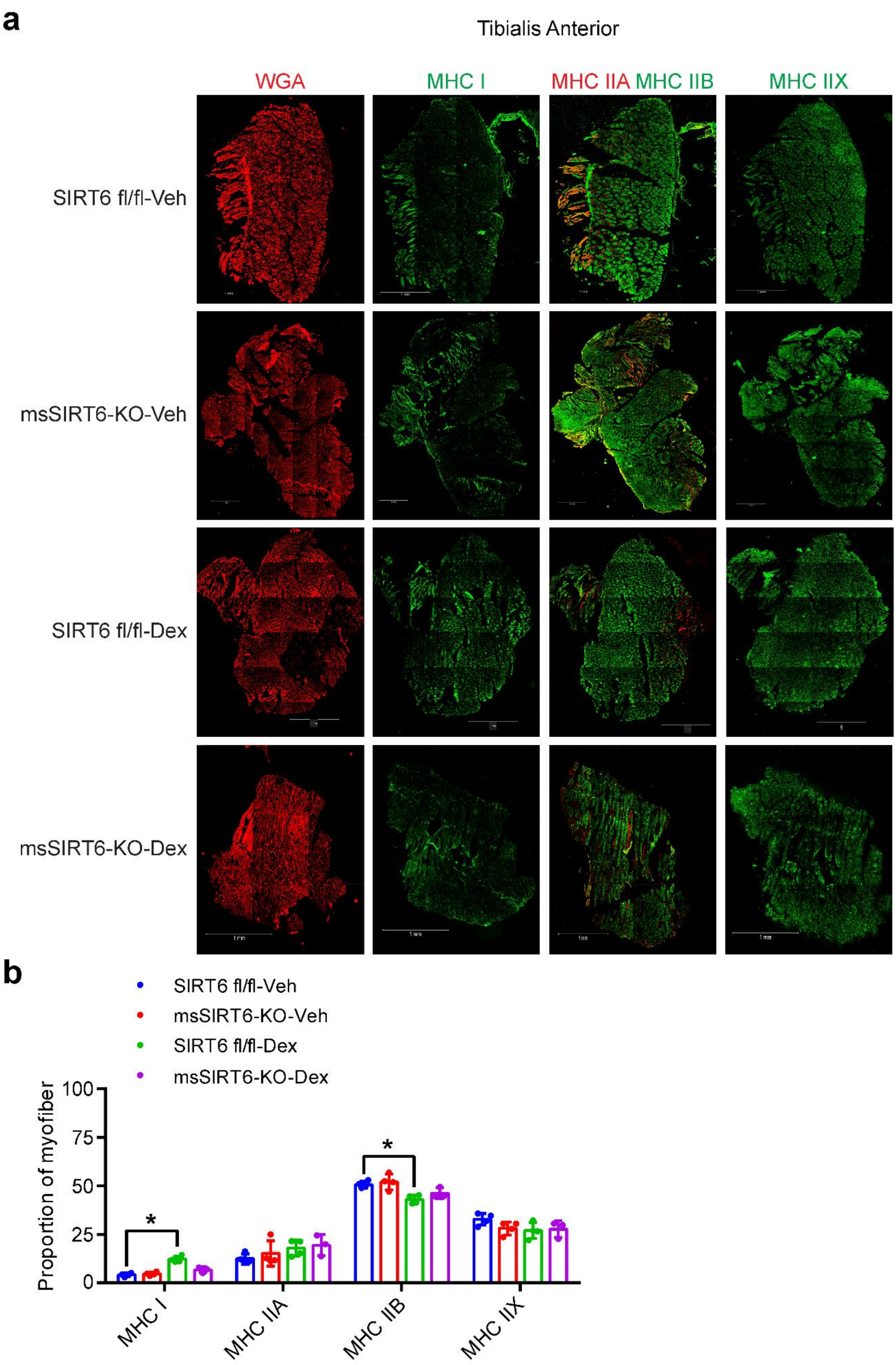

Supplementary Figure 13: Muscle-specific SIRT6 deficient mice are resistant to dexamethasone induced fiber type switching

a Representative image showing WGA (red) staining to determine CSA and MHC I (green), MHC IIA MHC IIB (MHC IIA; red, MHC IIB; green), MHC IIX (green) staining to determine proportion of muscle fiber types in Dex (10mg/kg/day) administered SIRT6-fl/fl and msSIRT6-KO mice TA muscle. Scale bar= 1 mm. SIRT6-fl/fl-Veh n = 4, SIRT6-fl/fl-Dex n = 4, msSIRT6-KO-Veh n = 4, msSIRT6-KO-Dex n = 3. **b** Quantification for proportion of MHC I, MHC IIA, MHC IIB and MHC IIX myofiber in mice described in **a**. SIRT6-fl/fl-Veh n = 4, SIRT6-fl/fl-Dex n = 4, msSIRT6-KO-Veh n = 4, msSIRT6-KO-Dex n = 3. Data presented as mean ± s.d, $^{*}p < 0.05$. Two-tailed unpaired Student's t-test was used for statistical analysis **b**. Source data are provided as a Source Data file.

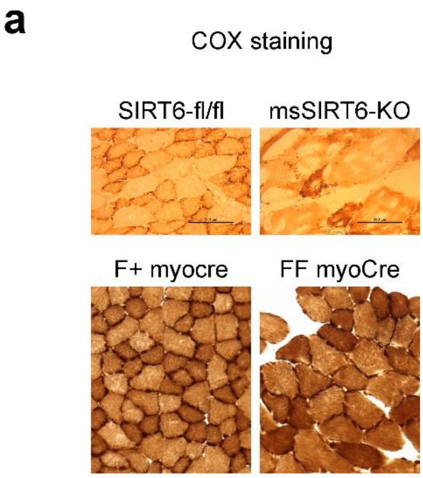

Supplementary Figure 14: Muscle-specific SIRT6 deficient mice muscle shows enlarged fast fiber types

a Representative images showing COX staining in SIRT6-fl/fl and msSIRT6-KO mice TA muscle (top) or F+myoCre and FF myoCre mice gastrocnemius muscle (bottom). Scale bar= 500 µm. SIRT6-fl/fl n = 4, msSIRT6-KO n = 4, F+myoCre n = 3, FF myoCre n = 3.

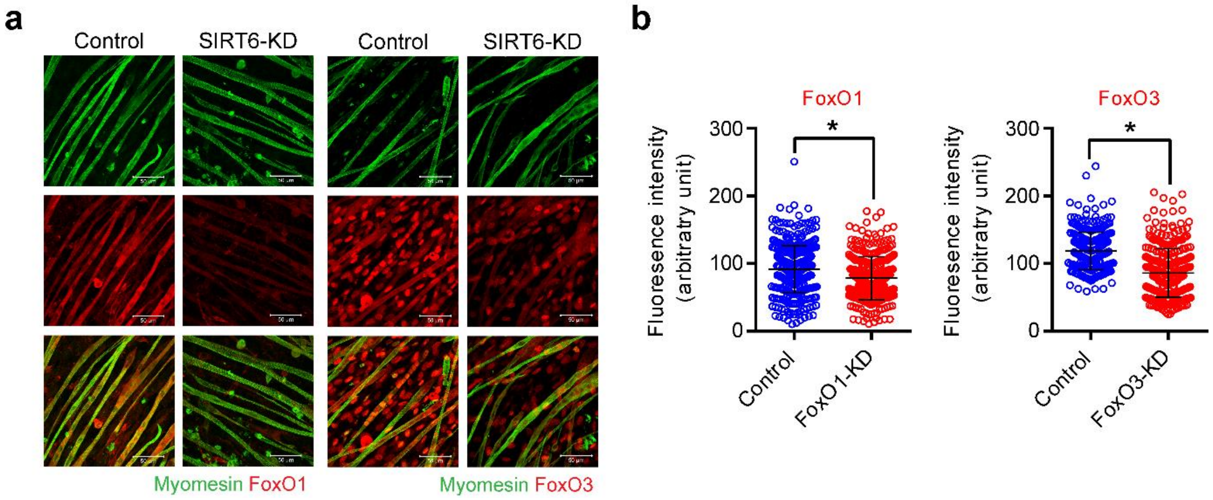

Supplementary Figure 15: Validation of siRNA mediated depletion of FoxO1 and FoxO3

a Representative confocal images of FoxO1 or FoxO3 levels to confirm RNAi-mediated depletion of FoxO1 (left) or FoxO3 (right) in primary myotube transfected with control siRNA or siRNA targeting FoxO1 or FoxO3. FoxO1 or FoxO3 were stained in red and myomesin in green. Scale bar = 50 µm. **b** Scatterplot showing FoxO1 (left) or FoxO3 (right) fluorescence intensity in myotubes described in **a**. FoxO1 (Control n = 425, FoxO1-KD n = 332), FoxO3 (Control n = 323, FoxO3-KD n = 345). Data presented as mean ± s.d, *p < 0.05. Two-tailed unpaired Student's t-test was used for statistical analysis **b**. Source data are provided as a Source Data file.

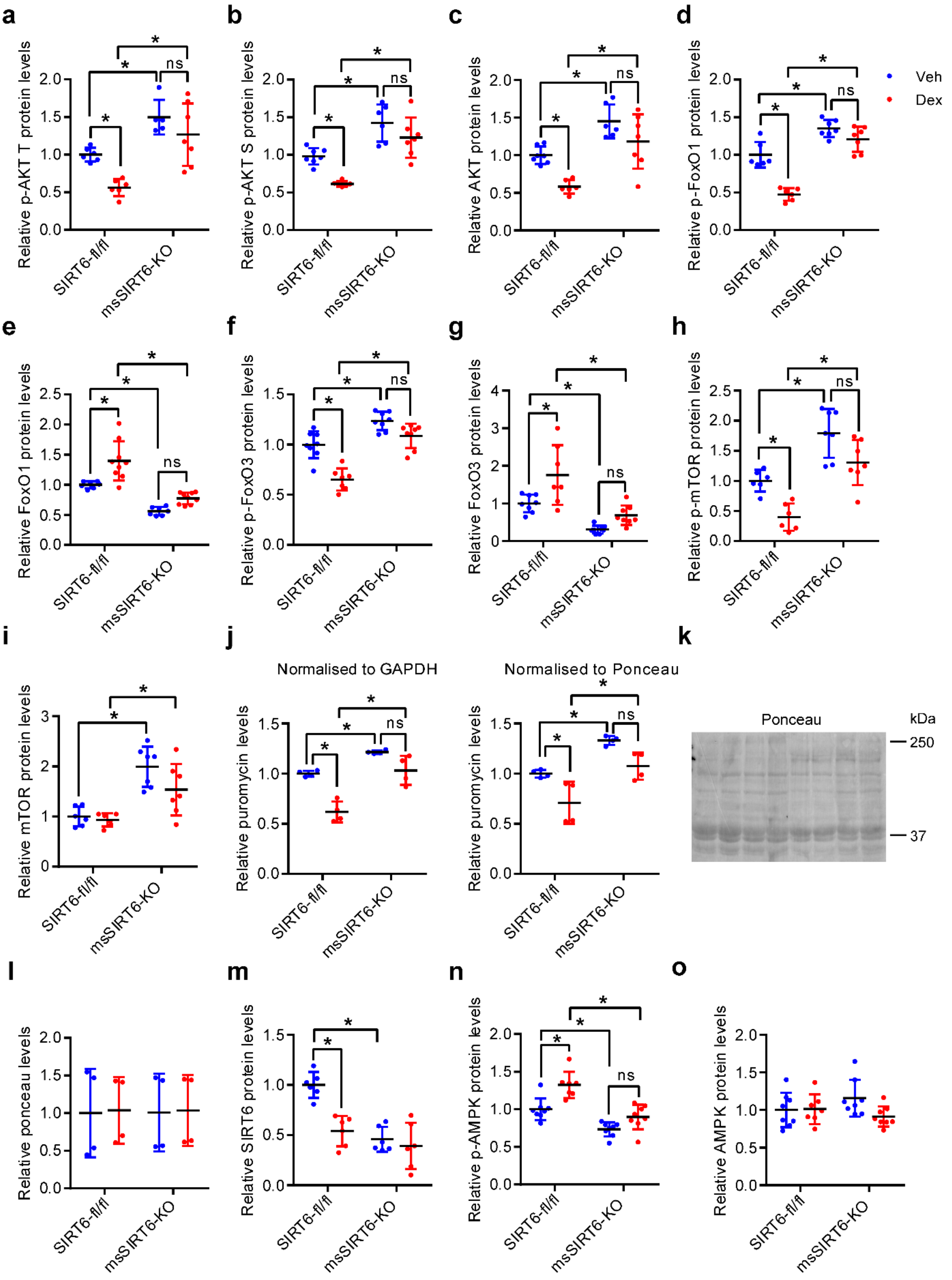

Supplementary Figure 16: SIRT6 regulates IGF/PI3K/AKT signaling in muscle

a Quantification of p-AKT T in Dex administered SIRT6-fl/fl, msSIRT6-KO mice. SIRT6-fl/fl-Veh, SIRT6-fl/fl-Dex n = 6, msSIRT6-KO-Veh n = 5, msSIRT6-KO-Dex n = 7. **b** Quantification

of p-AKT S in Dex administered SIRT6-fl/fl, msSIRT6-KO mice. SIRT6-fl/fl-Veh, msSIRT6-KO-Dex n = 7, SIRT6-fl/fl-Dex, msSIRT6-KO-Veh n = 6. **c** Quantification of AKT in Dex administered SIRT6-fl/fl, msSIRT6-KO mice. SIRT6-fl/fl-Veh, msSIRT6-KO-Dex n = 7, SIRT6-fl/fl-Dex, msSIRT6-KO-Veh n = 6. **d** Quantification of p-FoxO1 in Dex administered SIRT6-fl/fl, msSIRT6-KO mice. SIRT6-fl/fl-Veh, SIRT6-fl/fl-Dex n = 6, msSIRT6-KO-Veh, msSIRT6-KO-Dex n = 7. **e** Quantification of FoxO1 in Dex administered SIRT6-fl/fl, msSIRT6-KO mice. SIRT6-fl/fl-Veh, msSIRT6-KO-Veh n = 7, SIRT6-fl/fl-Dex, msSIRT6-KO-Dex n = 9. **f** Quantification of p-FoxO3 in Dex administered SIRT6-fl/fl, msSIRT6-KO mice. SIRT6-fl/fl-Veh, msSIRT6-KO-Veh, msSIRT6-KO-Dex n = 8, SIRT6-fl/fl-Dex, n = 7. **g** Quantification of FoxO3 in Dex administered SIRT6-fl/fl, msSIRT6-KO mice. SIRT6-fl/fl-Veh, msSIRT6-KO-Veh, msSIRT6-KO-Dex n = 8, SIRT6-fl/fl-Dex, n = 7. **h** Quantification of p-mTOR in Dex administered SIRT6-fl/fl, msSIRT6-KO mice. SIRT6-fl/fl-Veh, SIRT6-fl/fl-Dex n = 6, msSIRT6-KO-Veh, msSIRT6-KO-Dex n = 7. **i** Quantification of mTOR in Dex administered SIRT6-fl/fl, msSIRT6-KO mice. SIRT6-fl/fl-Veh, SIRT6-fl/fl-Dex n = 6, msSIRT6-KO-Veh, msSIRT6-KO-Dex n = 7. **j** Quantification of SUnSET analysis normalized to GAPDH (left) and normalized to ponceau (right) in Dex administered SIRT6-fl/fl, msSIRT6-KO mice. n = 4. **k** Ponceau stained western blot in Figure 9A. **l** Ponceau levels for puromycin western blot in Figure 9A. n = 4. **m** Quantification of SIRT6 in Dex administered SIRT6-fl/fl, msSIRT6-KO mice. n = 6. **n** Quantification of p-AMPK in Dex administered SIRT6-fl/fl, msSIRT6-KO mice. SIRT6-fl/fl-Veh, msSIRT6-KO-Veh, msSIRT6-KO-Dex n = 8, SIRT6-fl/fl-Dex n = 7. **o** Quantification of AMPK in Dex administered SIRT6-fl/fl, msSIRT6-KO mice. SIRT6-fl/fl-Veh, msSIRT6-KO-Veh, msSIRT6-KO-Dex n = 8, SIRT6-fl/fl-Dex n = 7. Data presented as mean ± s.d, *p < 0.05, ns – not significant. Two-way ANOVA with Bonferroni post hoc test was used for statistical analysis **a-j, l-o**. Gastrocnemius from Dex (10mg/kg/day) administered SIRT6-fl/fl and msSIRT6-KO mice was used for western analysis **a-o**. Protein were normalizing to GAPDH **a-i, j (left), m-o**. Source data are provided as a Source Data file.

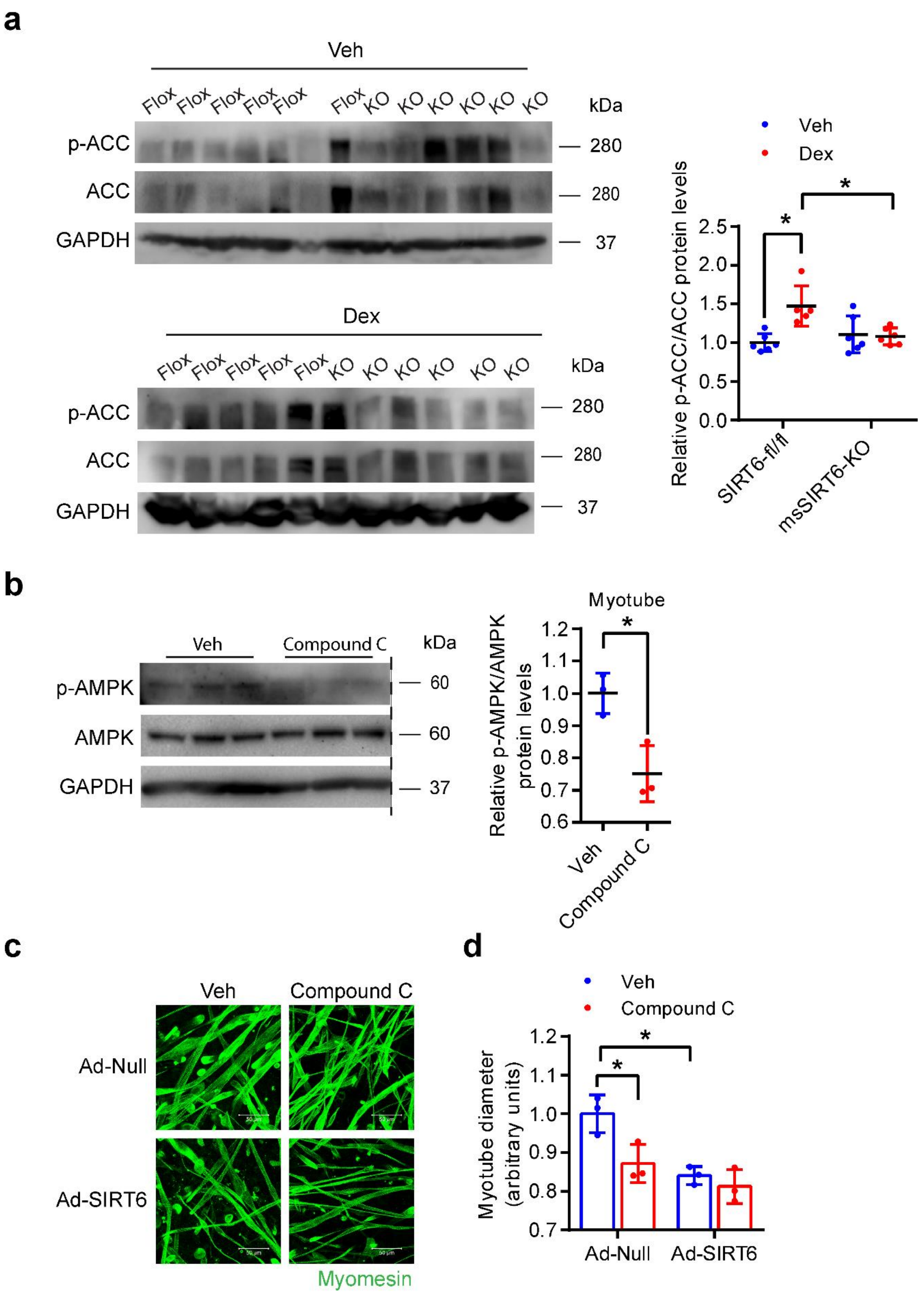

Supplementary Figure 17

Supplementary Figure 17: SIRT6 regulates myotube diameter independent of AMPK signaling

a Western blot (left)) and quantification (right) of p-ACC normalized to ACC in Dex (10mg/kg/day) or Veh administered SIRT6-fl/fl and msSIRT6-KO mice gastrocnemius muscle. SIRT6-fl/fl-Veh, msSIRT6-KO-Veh, and msSIRT6-KO-Dex n = 6, SIRT6-fl/fl-Dex n = 5. **b** Western blot (left) and quantification (right) of p-AMPK normalized to AMPK in AMPK inhibitor (Compound C) treated primary myotubes. n = 3. **c** Representative confocal images depicting diameter of SIRT6 overexpressing primary myotubes treated with either AMPK inhibitor (Compound C) or Veh. The myotubes were stained green using antibody against myomesin. Scale bar = 50 µm. **d** Quantification for fiber diameter in primary myotubes described in **c**. n = 3. Data presented as mean ± s.d, *p < 0.05, ns - not significant. Two-way ANOVA with Bonferroni post hoc test was used for statistical analysis **a, d**. Two-tailed unpaired Student's t-test was used for statistical analysis **b**. Source data are provided as a Source Data file.

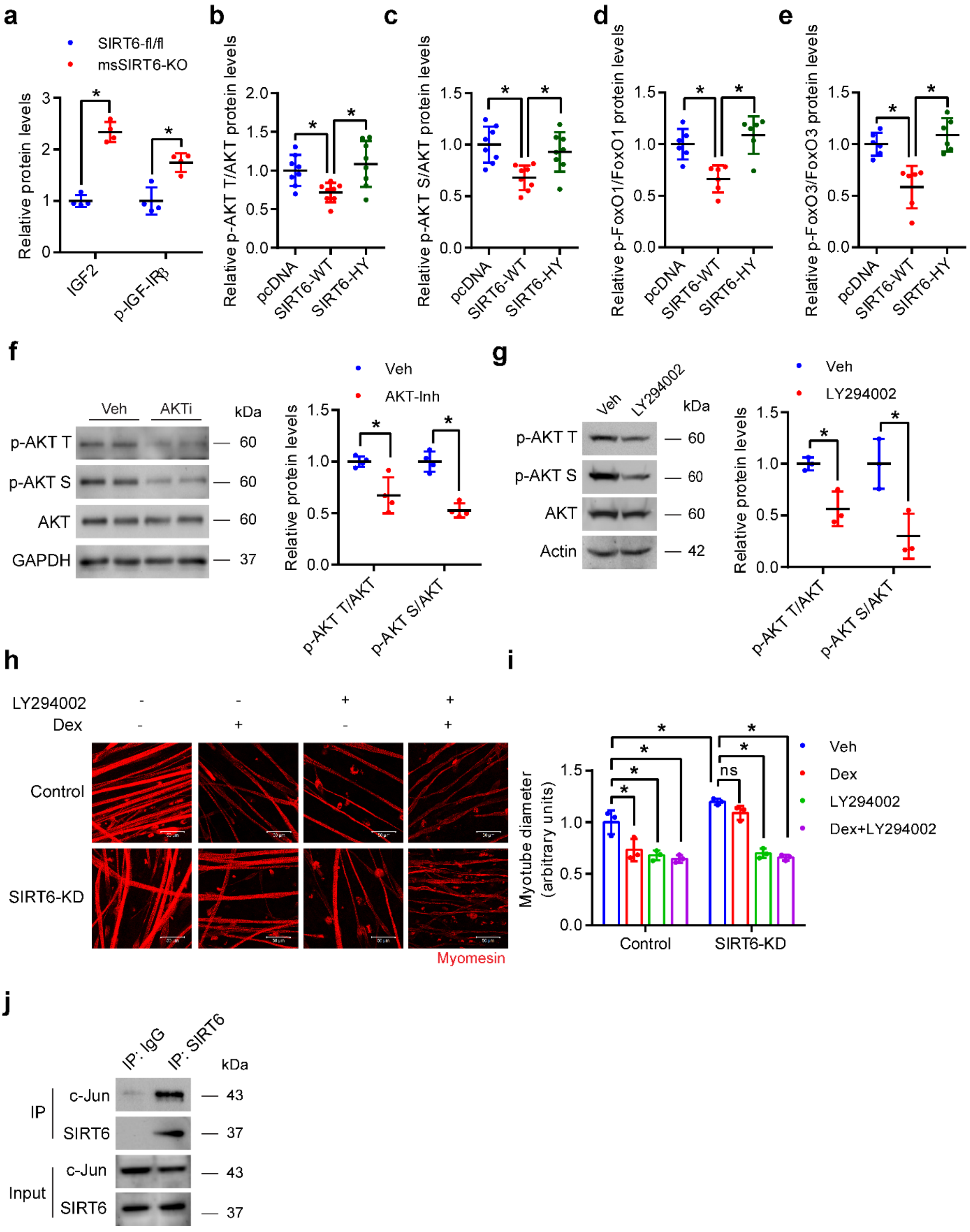

Supplementary Figure 18: SIRT6 regulates IGF/PI3K/AKT signaling in muscle in catalytic activity dependent manner

a Quantification of IGF2 and phosphorylated protein levels of IGF-I-Receptor β normalized to GAPDH in Dex (10mg/kg/day) administered SIRT6-fl/fl and msSIRT6-KO mice gastrocnemius muscle. n = 4. **b** Quantification of p-AKT T normalized to AKT in stable knockdown 293T cells

transfected with pcDNA, SIRT6-WT or SIRT6-H133Y plasmids. pcDNA, SIRT6-WT or SIRT6-H133Y n = 8. **c** Quantification of p-AKT S normalized to AKT in stable knockdown 293T cells transfected with pcDNA, SIRT6-WT or SIRT6-H133Y plasmids. pcDNA, SIRT6-WT or SIRT6-H133Y n = 8. **d** Quantification of p-FoxO1 normalized to FoxO1 in stable knockdown 293T cells transfected with pcDNA, SIRT6-WT or SIRT6-H133Y plasmids. pcDNA, SIRT6-WT or SIRT6-H133Y n = 6. **e** Quantification of p-FoxO3 normalized to FoxO3 in stable knockdown 293T cells transfected with pcDNA, SIRT6-WT or SIRT6-H133Y plasmids. pcDNA, SIRT6-WT or SIRT6-H133Y n = 6. **f** Western blot (left) and quantification (right) of p-AKT T and p-AKT S normalized to AKT in AKT inhibitor (AKTi-1/2) treated primary myotubes. n = 4. **g** Western blot (left) and quantification (right) of p-AKT T and p-AKT S normalized to AKT in PI3K inhibitor (LY294002) treated primary myotubes. n = 3. **h** Representative confocal images showing myotubes diameter in PI3K inhibitor (LY294002) and/or Dex treated SIRT6 depleted myotubes. The myotubes were stained red with antibody against myomesin. Scale bar = 50 µm. **i** Quantification of myotube diameter described in **h**. n = 3. **j** Western blotting images showing c-Jun interaction with SIRT6. SIRT6 was immunoprecipitated from primary myotubes and the interaction with c-Jun was tested by western blotting. n = 3. Data presented as mean ± s.d, *$p < 0.05$, Two-tailed unpaired Student's t-test was used for statistical analysis **a, f, g, i**. One-way ANOVA with Bonferroni post hoc test was used **b-e**. Source data are provided as a Source Data file.

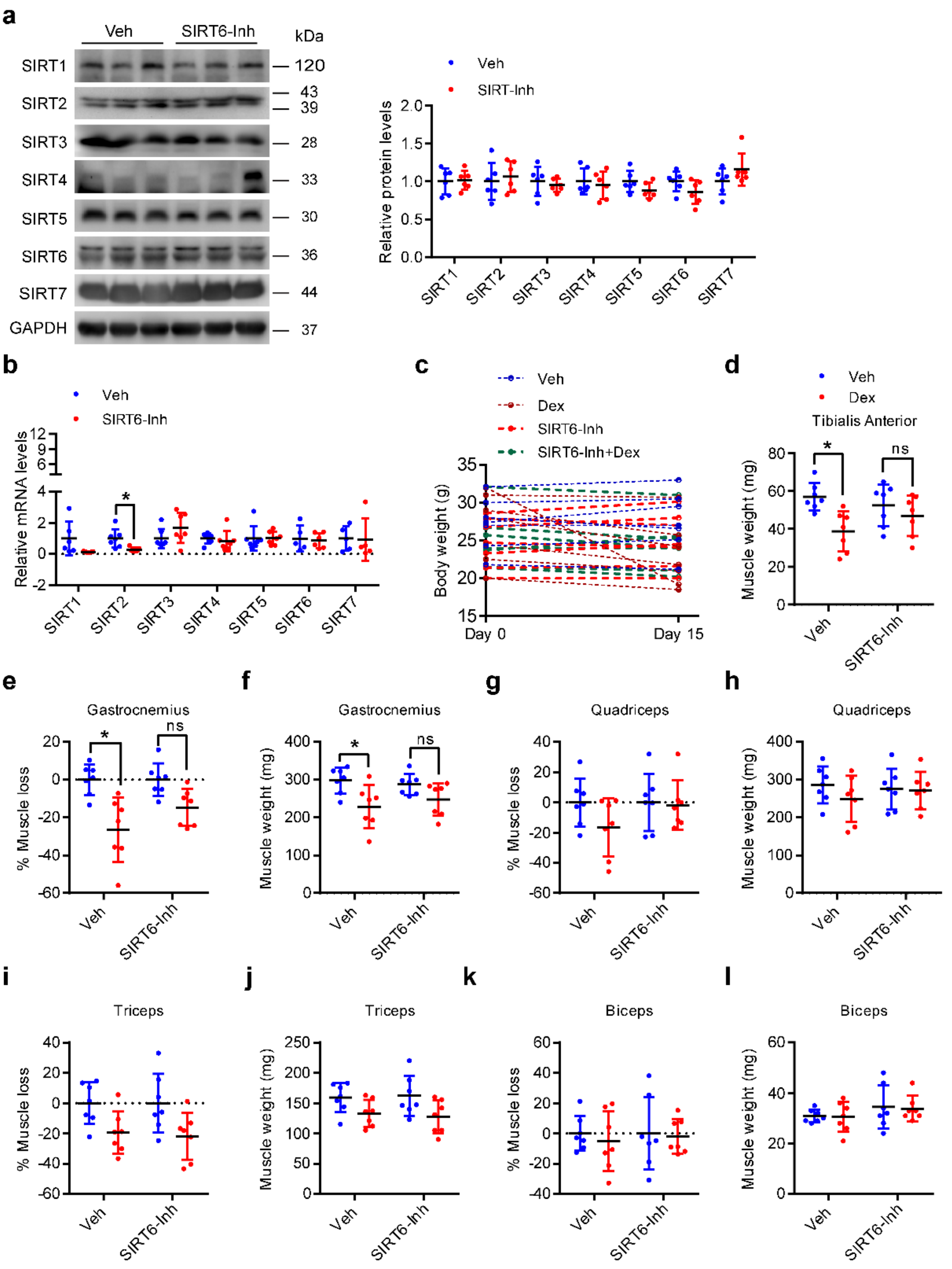

Supplementary Figure 19

Supplementary Figure 19: Pharmacological SIRT6 inhibition protects against dexamethasone induced muscle loss

a Western blot (left) and quantification (right) of protein levels of all Sirtuin isoforms in SIRT6 inhibitor (SIRT6-Inh) treated mice gastrocnemius muscle. n = 6. **b** qPCR analysis of relative mRNA levels of all Sirtuin isoforms in SIRT6-Inh treated mice gastrocnemius muscle. SIRT1 (Veh n = 6, SIRT6-Inh n = 7), SIRT2 (Veh, SIRT6-Inh n = 7), SIRT3 (Veh, SIRT6-Inh n = 7),

SIRT4 (Veh, SIRT6-Inh n = 7), SIRT5 (Veh, SIRT6-Inh n = 7), SIRT6 (Veh n = 5, SIRT6-Inh n = 6), SIRT7 (Veh, SIRT6-Inh n = 5). **c** Change in body weight with time for each Veh and SIRT6-Inh treated mouse after Dex injection. n = 7. **d** Loss in absolute tibialis anterior muscle mass from Veh and SIRT6-Inh treated mice after intraperitoneal Dex injection. n = 7. **e** Percentage (%) loss in gastrocnemius muscle mass from Veh and SIRT6-Inh treated mice after Dex injection. n = 7. **f** Loss in absolute gastrocnemius muscle mass from SIRT6-Inh treated mice after Dex injection. n = 7. **g** Percentage (%) loss in quadriceps muscle mass from SIRT6-Inh treated mice after Dex injection. n = 7. **h** Loss in absolute quadriceps muscle mass from SIRT6-Inh treated mice after Dex injection. n = 7. **i** Percentage (%) loss in triceps muscle mass from SIRT6-Inh treated mice after Dex injection. n = 7. **j** Loss in absolute triceps muscle mass from SIRT6-Inh treated mice after Dex injection. n = 7. **k** Percentage (%) loss in biceps muscle mass from SIRT6-Inh treated mice after Dex injection. n = 7. **l** Loss in absolute biceps muscle mass from SIRT6-Inh treated mice after Dex injection. n = 7. Data presented as mean ± s.d, ns – not significant. Two-tailed unpaired Student's t-test was used for statistical analysis **a, b**. Two-way ANOVA with Bonferroni post hoc test was used for statistical analysis **d-l**. Mice were administered with SIRT6 inhibitor (15mg/kg/day) or Dex (20mg/kg/day) **a-l**. Source data are provided as a Source Data file.

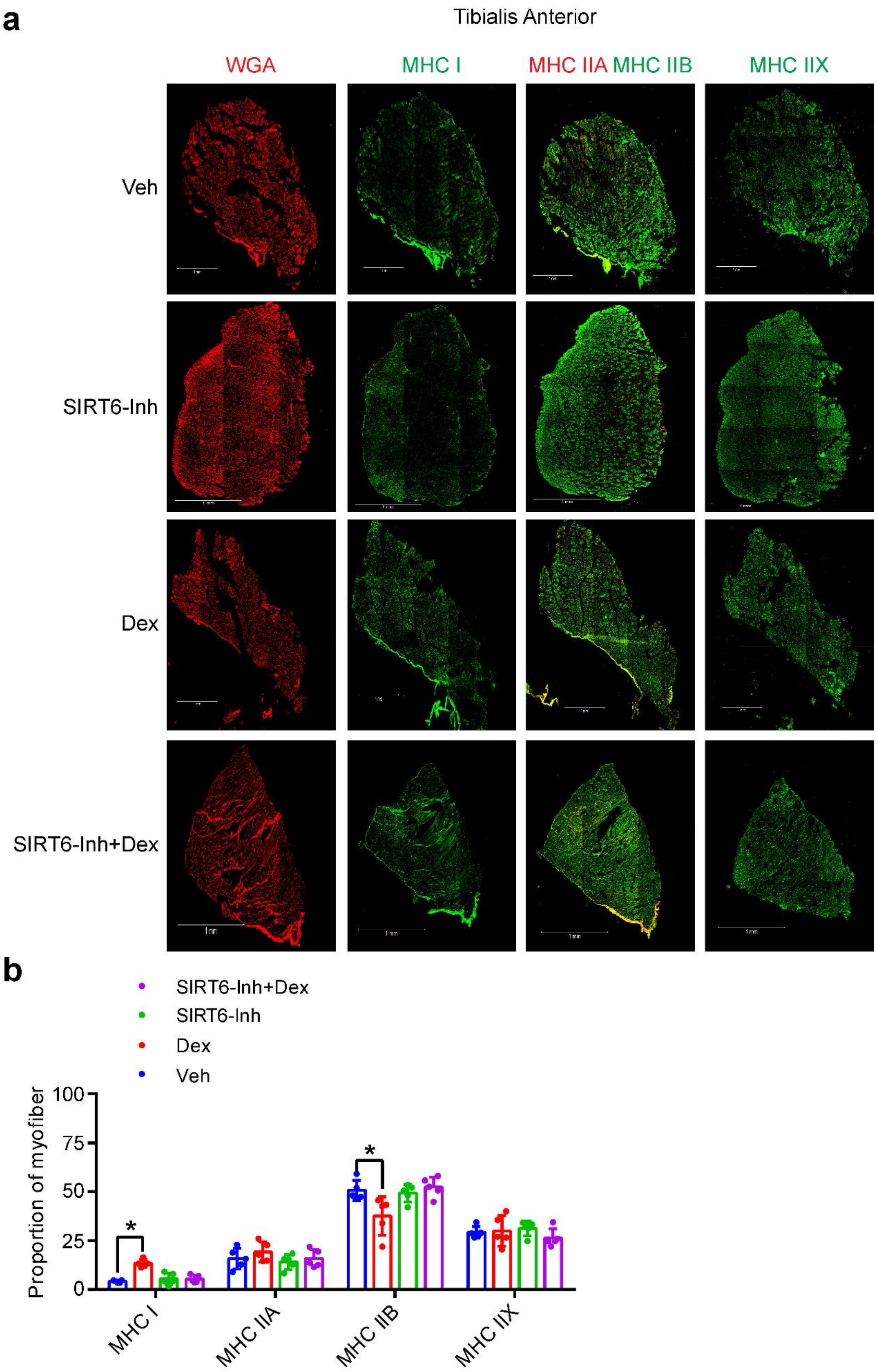

Supplementary Figure 20: Pharmacological SIRT6 inhibition protects against dexamethasone induced fiber type switching

a Representative images showing WGA (red) staining to determine CSA and MHC I (green), MHC IIA MHC IIB (MHC IIA; red, MHC IIB; green), MHC IIX (green) staining to determine proportion of muscle fiber types in Dex (20mg/kg/day) administered SIRT6-Inh (15mg/kg/day) treated mice TA muscle. Scale bar= 1 mm. n = 5. **b** Quantification for proportion of MHC I, MHC IIA, MHC IIB and MHC IIX myofiber in mice described in **a**. n = 5. Data presented as mean ± s.d, *p < 0.05. Two-tailed unpaired Student's t-test was used for statistical analysis. **b** Source data are provided as a Source Data file.

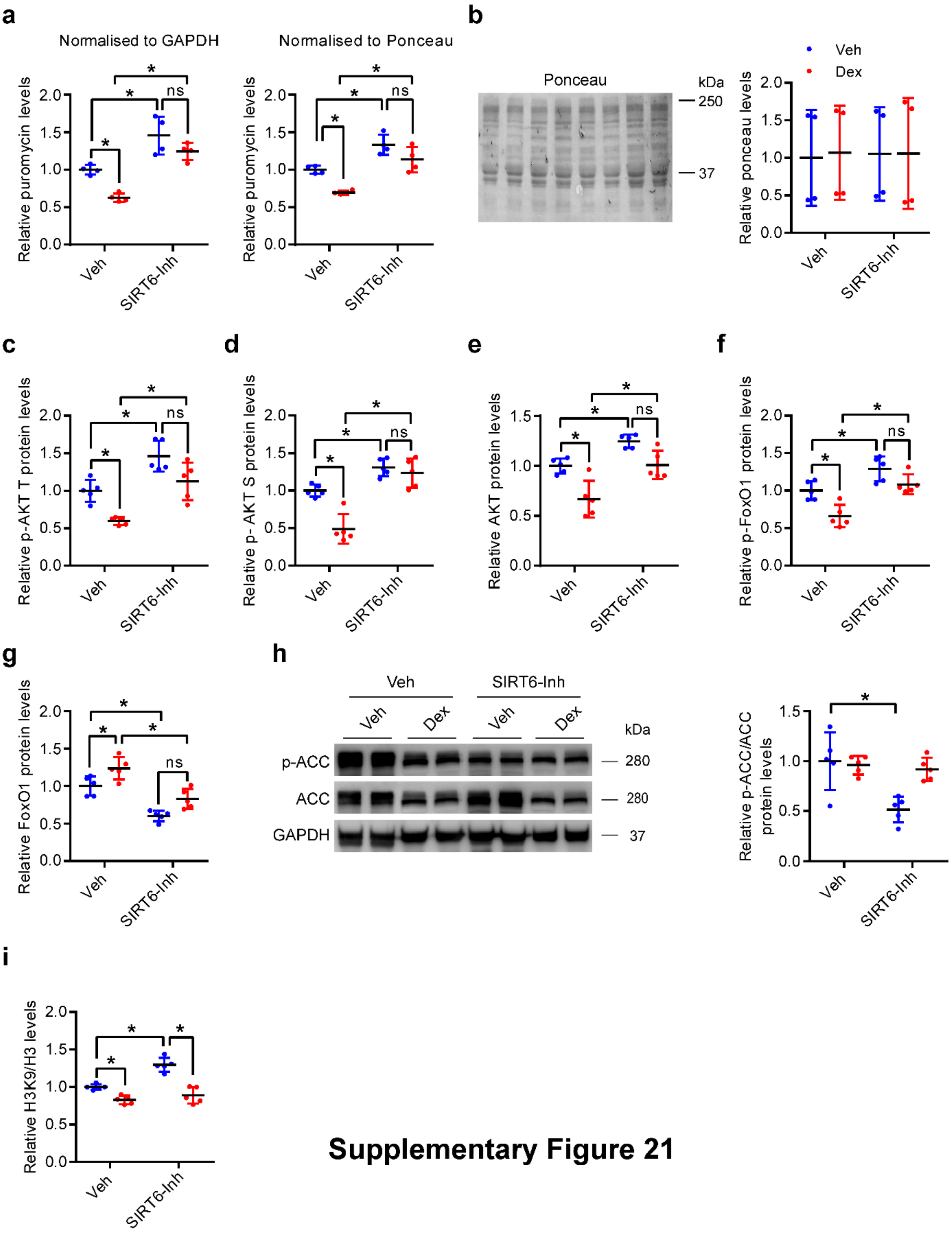

Supplementary Figure 21

Supplementary Figure 21: Pharmacological SIRT6 inhibition hyperactivates IGF/PI3K/AKT signaling in muscle

a Quantification of SUnSET analysis of protein synthesis normalized to GAPDH (left) and normalized to ponceau (right) in Dex administered SIRT6-Inh treated mice gastrocnemius muscle. n = 4. **b** Ponceau stained western blot (left) and quantification of ponceau staining

(right) showing protein equal loading for SUnSET analysis of protein synthesis in Figure 10G. n = 4. **c** Quantification of p-AKT T normalized to GAPDH in Dex administered SIRT6-Inh treated mice gastrocnemius muscle. n = 5. **d** Quantification of p-AKT S normalized to GAPDH in Dex administered SIRT6-Inh treated mice gastrocnemius muscle. n = 5. **e** Quantification of AKT normalized to GAPDH in Dex administered SIRT6-Inh treated mice gastrocnemius muscle. n = 5. **f** Quantification of p-FoxO1 normalized to GAPDH in Dex administered SIRT6-Inh treated mice gastrocnemius muscle. n = 5. **g** Quantification of FoxO1 normalized to GAPDH in Dex administered SIRT6-Inh treated mice gastrocnemius muscle. n = 5. **h** Western blot (left) and quantification of p-ACC normalized to ACC in Dex administered SIRT6-Inh treated mice gastrocnemius muscle. n = 5. **i** Quantification of global acetylation of histone 3 at lysine 9 normalized to histone 3 in Dex administered SIRT6 inhibitor treated mice gastrocnemius muscle. n = 5. Data presented as mean ± s.d, *p < 0.05, ns - not significant. Two-way ANOVA with Bonferroni post hoc test was used for statistical analysis **a-i**. Mice were administered with SIRT6 inhibitor (15mg/kg/day) or Dex (20mg/kg/day) **a-i**. Source data are provided as a Source Data file.

Supplementary Tables

Supplementary Table 1: Absolute muscle weight and tibia length of Dex administered mice

Group	Veh (Day 1)	Dex (Day 1)	Veh (Day 7)	Dex (Day 7)	Veh (Day 15)	Dex (Day 15)
Tibialis anterior	95.8±22.4	92.8±18.1	93.9±18.9	81.3±16.0	97.4±12.1	72.4±15.4
Gastrocnemius	264.8±25.3	263.3±45.1	263.4±29.3	252.0±24.4	257.4±44.7	199.3±44.9
Soleus	15.2±0.7	14.5±2.2	14.1±2.1	12.9±3.2	13.5±2.4	10.1±2.9
Quadriceps	300±64.0	283.0±60.1	287.4±46.0	255.6±30.4	284.9±70.7	235.3±43.3
Triceps	197±46.4	160.7±34.7	157.3±22.2	158.0±26.1	159.8±30.7	145.4±26.0
Biceps	45.6±12.8	38.0±12.1	33.4±3.1	33.3±5.8	39.0±9.3	41.0±10.8
Tibia length	19.78±0.9	19.1±0.5	18.0±0.4	18.1±0.3	17.8±0.6	17.6±0.9

Supplementary Table 2: Absolute muscle weight and tibia length of Dex administered msSIRT6-KO mice

Group	SIRT6-fl/fl-Veh	SIRT6-fl/fl-Dex	msSIRT6-KO-Veh	msSIRT6-KO-Dex
Tibialis anterior	94.6±32.8	53.5±12.4	106.1±11.6	77.7±18.9
Gastrocnemius	278.2±51.1	188.0±52.1	314.3±44.8	247.9±43.8
Quadriceps	462.8±83.6	382.7±71.3	461.1±84.0	404.8±51.8
Triceps	206.9±36.8	178.9±33.3	196.6±40.3	164.4±20.0
Biceps	28.6±8.4	27.2±6.9	33.3±8.5	32.5±7.8
Tibia length	20.8±0.6	20.0±0.6	20.6±1.0	20.3±0.8

Supplementary Table 3: Absolute muscle weight and tibia length of Dex administered SIRT6 inhibitor treated mice

Group	Veh	Dex	SIRT6-Inh	SIRT6-Inh -Dex
Tibialis anterior	57.0±6.7	38.7±9.9	52.4±10.3	47.0±10.0
Gastrocnemius	297.7±31.6	228.3±52.5	287.4±25.7	247.3±40.4
Quadriceps	286.1±45.2	249.1±56.8	275.0±50.2	271.1±45.7
Triceps	159.1±22.5	133.3±20.6	162.0±30.7	127.0±25.4
Biceps	30.9±2.4	30.6±5.6	34.4±8.0	33.9±4.6
Tibia length	18.0±0.8	18.8±0.8	17.9±0.3	18.0±1.5

Supplementary Table 4: Gradient profile for HPLC setting

Time (min)	Mobile phase A%	Mobile phase B%
0	100	0
0.5	98	2
7	87	13
10	68	32
30	0	100
50	100	0
55	100	0

Supplementary Table 5: List of antibodies and their dilution used in the study

Name	Application (Dilution)	Source and clone	Catalogue Number /Identifier
Puromycin	WB (1:500)	Developmental Studies Hybridoma Bank, 4A12	PMY-2A4
SIRT6	WB (1:1000)	Cell Signaling, D8D12	12486
p-AKT S	WB (1:1000)	Cell Signaling, D9E	4060
p-AKT T	WB (1:1000)	Cell Signaling, D25E6	13038
AKT	WB (1:1000)	Cell Signaling, C67E7	4691
p-FoxO1	WB (1:1000)	Cell Signaling	9461
p-FoxO3	WB (1:1000)	Cell Signaling, D18H8	13129
p-mTOR	WB (1:1000)	Cell Signaling, D9C2	5536
p-AMPK	WB (1:500)	Cell Signaling, 40H9	2535
p-ACC	WB (1:1000)	Cell Signaling, D7D11	11818
mTOR	WB (1:1000)	Cell Signaling, 7C10	2983
AMPK	WB (1:1000)	Cell Signaling	2532
ACC	WB (1:1000)	Cell Signaling, C83B10	3676
Acetyl-Histone H3 (Lys9)	WB (1:1000)	Cell Signaling, C5B11	9649
Acetyl-Histone H3 (Lys18)	WB (1:1000)	Cell Signaling	9675
Histone H3	WB (1:1000)	Cell Signaling	9715
Anti-SOD2/MnSOD (acetyl K68)	WB (1:1000)	Abcam, EPVANR2	ab137037
Anti-SOD2/MnSOD	WB (1:1000)	Abcam	ab13533
SIRT1	WB (1:500)	Cloud-Clone	PAE912Mu01
SIRT2	WB (1:1000)	Sigma-Aldrich	S8447
SIRT3	WB (1:1000)	Cell Signaling, D22A3	5490
SIRT4	WB (1:500)	Cloud-Clone	PAE914Mu01
SIRT5	WB (1:1000)	Cell Signaling, D8C3	8782
SIRT7	WB (1:500)	Cloud-Clone	PAE917Hu01
c-Jun	WB (1:500)	Santa Cruz	sc-1694
GAPDH	WB (1:1000)	Santa Cruz	sc-25778
Actin	WB (1:5000)	Sigma-Aldrich, AC-15	A3854

Vinculin	WB (1:1000)	Cell Signaling, E1E9V	13901
Myomesin	IF (1:50)	Developmental Studies Hybridoma Bank, mMaC myomesin B4	mMaC myomesin B4
Atrogin-1	IF (1:100)	Abcam	ab74023
MuRF-1/2/3	IF (1:100)	Abcam, EPR6431(2)	Ab172479
FoxO1	IF (1:200) WB (1:1000)	Cell Signaling, C29H4	2880
FoxO3	IF (1:200) WB (1:1000)	Cell Signaling, D19A7	12829
Myosin heavy chain Type I	IHC (1:5)	Developmental Studies Hybridoma Bank, BA-D5	BA-D5
Myosin heavy chain Type IIA	IHC (1:5)	Developmental Studies Hybridoma Bank, SC-71	SC-71
Myosin heavy chain Type IIB	IHC (1:5)	Developmental Studies Hybridoma Bank, BF-F3	BF-F3
Myosin heavy chain Type IIX	IHC (1:5)	Developmental Studies Hybridoma Bank, 6H1	6H1
Normal Rabbit IgG	ChIP	Cell Signaling	2729
Anti-Rabbit HRP	WB (1:5000)	Cell Signaling	7074
Anti-Mouse HRP	WB (1:5000)	Cell Signaling	7076
Clean-Blot IP Detection Reagent	WB (1:2000)	Thermo Fisher Scientific	21230
anti-Rabbit IgG light chain HRP	WB (1:1000)	Cell Signaling, D3V2A	58802
Donkey anti-Mouse IgG (H+L), Alexa Fluor 488	IF (1:400)	Thermo Fisher Scientific	A-21202
Donkey anti-Rabbit IgG (H+L), DyLight 594	IF (1:400)	Thermo Fisher Scientific	SA5-10040
Goat anti-Rabbit IgG (H+L), Alexa Fluor 546	IF (1:400)	Thermo Fisher Scientific	A-11035
Goat anti-Mouse IgG (H+ L), Alexa Fluor 488	IF (1:400)	Thermo Fisher Scientific	A-11001
Goat anti-Mouse IgG1, Alexa Fluor 594	IHC (1:5)	Thermo Fisher Scientific	A-21125

Goat anti-Mouse IgM (Heavy chain), Alexa Fluor 488	IHC (1:5)	Thermo Fisher Scientific	A-21042
