# Supplementary material for: Sirtuin 6 inhibition protects against glucocorticoid-induced skeletal muscle atrophy by regulating IGF/PI3K/AKT signaling
Source: Nat Commun. 2022 Sep 15;13:5415. doi: 10.1038/s41467-022-32905-w (PMC9478160; doi:10.1038/s41467-022-32905-w)
Supplement: Supplementary file 2 — Reporting Summary [file 41467_2022_32905_MOESM2_ESM.pdf]

nature portfolio

Corresponding author(s): Raul Mostoslavsky, Nagalingam R. Sundaresan

Last updated by author(s): Aug 5, 2022

Reporting Summary

Nature Portfolio wishes to improve the reproducibility of the work that we publish. This form provides structure for consistency and transparency in reporting. For further information on Nature Portfolio policies, see our Editorial Policies and the Editorial Policy Checklist.

Statistics

For all statistical analyses, confirm that the following items are present in the figure legend, table legend, main text, or Methods section.

n/a	Confirmed	
☐	☒	The exact sample size (*n*) for each experimental group/condition, given as a discrete number and unit of measurement
☒	☐	A statement on whether measurements were taken from distinct samples or whether the same sample was measured repeatedly
☐	☒	The statistical test(s) used AND whether they are one- or two-sided<br>*Only common tests should be described solely by name; describe more complex techniques in the Methods section.*
☒	☐	A description of all covariates tested
☒	☐	A description of any assumptions or corrections, such as tests of normality and adjustment for multiple comparisons
☐	☒	A full description of the statistical parameters including central tendency (e.g. means) or other basic estimates (e.g. regression coefficient) AND variation (e.g. standard deviation) or associated estimates of uncertainty (e.g. confidence intervals)
☐	☒	For null hypothesis testing, the test statistic (e.g. *F*, *t*, *r*) with confidence intervals, effect sizes, degrees of freedom and *P* value noted<br>*Give P values as exact values whenever suitable.*
☒	☐	For Bayesian analysis, information on the choice of priors and Markov chain Monte Carlo settings
☒	☐	For hierarchical and complex designs, identification of the appropriate level for tests and full reporting of outcomes
☒	☐	Estimates of effect sizes (e.g. Cohen's *d*, Pearson's *r*), indicating how they were calculated

Our web collection on statistics for biologists contains articles on many of the points above.

Software and code

Policy information about availability of computer code

Data collection	Confocal images were processed using Zen 2.1 Black software and quantification was done using ImageJ version 1.53c software. ImageJ version 1.53c software was used for quantification of myotube diameter, cross-sectional area of muscle fibers, mean fluorescence intensity and nuclear localization of FoxO and densitometric analysis of western blots.
Data analysis	Data analysis and graph preparation were performed using Graph-pad prism (Version 6.04).

For manuscripts utilizing custom algorithms or software that are central to the research but not yet described in published literature, software must be made available to editors and reviewers. We strongly encourage code deposition in a community repository (e.g. GitHub). See the Nature Portfolio guidelines for submitting code & software for further information.

Data

Policy information about availability of data

All manuscripts must include a data availability statement. This statement should provide the following information, where applicable:
- Accession codes, unique identifiers, or web links for publicly available datasets
- A description of any restrictions on data availability
- For clinical datasets or third party data, please ensure that the statement adheres to our policy

Source data supporting the findings in this study underlying dot plots, bar graphs, and uncropped western blots are provided in the online source data file. Source data are provided with this paper. We have neither used any publicly available datasets nor deposited dataset in any repository for this study.

Field-specific reporting

Please select the one below that is the best fit for your research. If you are not sure, read the appropriate sections before making your selection.

[x] Life sciences   [ ] Behavioural & social sciences   [ ] Ecological, evolutionary & environmental sciences

For a reference copy of the document with all sections, see nature.com/documents/nr-reporting-summary-flat.pdf

Life sciences study design

All studies must disclose on these points even when the disclosure is negative.

Sample size	No sample size calculation was performed using any statistical method. Sufficient sample size for in vitro and in vivo studies was determined based on previous lab experiences and research articles in the field (PMID: 33227539, 15125842, 31320633, 25834726) allowing to draw a robust conclusion.
Data exclusions	No data is excluded from analysis.
Replication	All attempts were made to generate reproducible data. We even cross verified the validity of data independently by two mice models from two independent labs. We have counted a large number of myotubes in the in vitro experiments to test the reproducibility and robustness of data which were repeated three or more independent times. We strongly believe our data will be reproducible.
Randomization	All the samples were randomized.
Blinding	Blinding was not relevant to planning of the experiments since we used mice models that required genotype assessment and subsequent experimentation. The investigators were blinded to group allocation while collecting and analysing the data.

Reporting for specific materials, systems and methods

We require information from authors about some types of materials, experimental systems and methods used in many studies. Here, indicate whether each material, system or method listed is relevant to your study. If you are not sure if a list item applies to your research, read the appropriate section before selecting a response.

Materials & experimental systems

n/a	Involved in the study	
[ ]	[x]	Antibodies
[ ]	[x]	Eukaryotic cell lines
[x]	[ ]	Palaeontology and archaeology
[ ]	[x]	Animals and other organisms
[x]	[ ]	Human research participants
[x]	[ ]	Clinical data
[x]	[ ]	Dual use research of concern

Methods

n/a	Involved in the study	
[x]	[ ]	ChIP-seq
[x]	[ ]	Flow cytometry
[x]	[ ]	MRI-based neuroimaging

Antibodies

Antibodies used

Details for antibodies used in western blotting (WB), immunofluorescence (IF) , immunohistochemistry (IHC) are provided provided in the supplementary file and also below:
WB: Puromycin (1:500) Developmental Studies Hybridoma Bank, clone 4A12, PMY-2A4
WB: SIRT6 (1:1000) Cell Signaling, clone D8D12, 12486
WB: p-AKT S (1:1000) Cell Signaling, clone D9E, 4060
WB: p-AKT T (1:1000) Cell Signaling, clone D25E6, 13038
WB: AKT (1:1000) Cell Signaling, clone C67E7, 4691
WB: p-FoxO1 (1:1000) Cell Signaling, 9461
WB: p-FoxO3 (1:1000) Cell Signaling, clone D18H8, 13129
WB: p-mTOR (1:1000) Cell Signaling, clone D9C2, 5536
WB: p-AMPK (1:500) Cell Signaling, clone 40H9, 2535
WB: p-ACC (1:1000) Cell Signaling, clone D7D11, 11818
WB mTOR (1:1000) Cell Signaling, clone 7C10, 2983
WB: AMPK (1:1000) Cell Signaling, 2532
WB: ACC (1:1000) Cell Signaling, clone C83B10, 3676
WB: Acetyl-Histone H3 (Lys9) (1:1000) Cell Signaling, clone C5B11, 9649
WB: Acetyl-Histone H3 (Lys18) (1:1000) Cell Signaling, 9675
WB: Histone H3 (1:1000) Cell Signaling, 9715
WB: Anti-SOD2/MnSOD (acetyl K68) (1:1000) Abcam, clone EPVANR2, ab137037

WB: Anti-SOD2/MnSOD (1:1000) Abcam, ab13533
WB: SIRT1 (1:500) Cloud-Clone, PAE912Mu01
WB: SIRT2 (1:1000) Sigma-Aldrich, S8447
WB: SIRT3 (1:1000) Cell Signaling, clone D22A3, 5490
WB: SIRT4 (1:500) Cloud-Clone, PAE914Mu01
WB: SIRT5 (1:1000) Cell Signaling, clone D8C3, 8782
WB: SIRT7 (1:500) Cloud-Clone, PAE917Hu01
WB: c-Jun (1:500) Santa Cruz, sc-1694
WB: GAPDH (1:1000) Santa Cruz, sc-25778
WB: Actin (1:5000) Sigma-Aldrich, clone AC-15, A3854
WB: Vinculin (1:1000) Cell Signaling, clone E1E9V, 13901
WB: FoxO1 (1:1000) Cell Signaling, clone C29H4, 2880
WB: FoxO3 (1:1000) Cell Signaling, clone D19A7, 12829
WB: Anti-Rabbit HRP (1:5000) Cell Signaling, 7074
WB: Anti-Mouse HRP (1:5000) Cell Signaling, 7076
WB: Clean-Blot IP Detection Reagent (1:2000) Thermo Fisher Scientific, 21230
WB: anti-Rabbit IgG light chain HRP (1:1000) Cell Signaling, clone D3V2A, 58802
IF: Myomesin (1:50) Developmental Studies Hybridoma Bank, clone mMaC myomesin, B4 mMaC myomesin B4
IF: Atrogin-1 (1:100) Abcam, ab74023
IF: MuRF-1/2/3 (1:100) Abcam, clone EPR6431(2), Ab172479
IF: FoxO1 (1:200) Cell Signaling, clone C29H4, 2880
IF: FoxO3 (1:200) Cell Signaling, clone D19A7, 12829
IF: Donkey anti-Mouse IgG (H+L), Alexa Fluor 488 (1:400) Thermo Fisher Scientific, A-21202
IF: Donkey anti-Rabbit IgG (H+L), DyLight 594 (1:400) Thermo Fisher Scientific, SA5-10040
IF: Goat anti-Rabbit IgG (H+L), Alexa Fluor 546 (1:400) Thermo Fisher Scientific, A-11035
IF: Goat anti-Mouse IgG (H+ L), Alexa Fluor 488 (1:400) Thermo Fisher Scientific, A-11001
IHC: Myosin heavy chain Type I (1:5) Developmental Studies Hybridoma Bank, clone BA-D5, BA-D5
IHC: Myosin heavy chain Type IIA (1:5) Developmental Studies Hybridoma Bank, clone SC-71, SC-71
IHC: Myosin heavy chain Type IIB (1:5) Developmental Studies Hybridoma Bank, clone BF-F3, BF F3
IHC: Myosin heavy chain Type IIX IHC (1:5) Developmental Studies Hybridoma Bank, clone 6H1, 6H1
IHC: Goat anti-Mouse IgG1, Alexa Fluor 594 (1:5) Thermo Fisher Scientific, A-21125
IHC: Goat anti-Mouse IgM (Heavy chain), Alexa Fluor 488 (1:5) Thermo Fisher Scientific, A-21042

Validation

SIRT6 antibody was validated using KO cell lines. All the other antibodies used in the study were commercial available and specificity was validated by manufacturers. Below are the details of the same.
Puromycin, https://dshb.biology.uiowa.edu/PMY-2A4
SIRT6, https://www.cellsignal.com/products/primary-antibodies/sirt6-d8d12-rabbit-mab/12486
p-AKT S, https://www.cellsignal.com/products/primary-antibodies/phospho-akt-ser473-d9e-xp-rabbit-mab/4060
p-AKT T, https://www.cellsignal.com/products/primary-antibodies/phospho-akt-thr308-d25e6-xp-rabbit-mab/13038
AKT, https://www.cellsignal.com/products/primary-antibodies/akt-pan-c67e7-rabbit-mab/4691
p-FoxO1, https://www.cellsignal.com/products/primary-antibodies/phospho-foxo1-ser256-antibody/9461
p-FoxO3, https://www.cellsignal.com/products/primary-antibodies/phospho-foxo3a-ser253-d18h8-rabbit-mab/13129
p-mTOR, https://www.cellsignal.com/products/primary-antibodies/phospho-mtor-ser2448-d9c2-xp-rabbit-mab/5536
p-AMPK, https://www.cellsignal.com/products/primary-antibodies/phospho-ampka-thr172-40h9-rabbit-mab/2535
p-ACC, https://www.cellsignal.com/products/primary-antibodies/phospho-acetyl-coa-carboxylase-ser79-d7d11-rabbit-mab/11818
mTOR, https://www.cellsignal.com/products/primary-antibodies/mtor-7c10-rabbit-mab/2983
AMPK, https://www.cellsignal.com/products/primary-antibodies/ampka-antibody/2532
ACC, https://www.cellsignal.com/products/primary-antibodies/acetyl-coa-carboxylase-c83b10-rabbit-mab/3676
Acetyl-Histone H3 (Lys9), https://www.cellsignal.com/products/primary-antibodies/acetyl-histone-h3-lys9-c5b11-rabbit-mab/9649
Acetyl-Histone H3 (Lys18), https://www.cellsignal.com/products/primary-antibodies/acetyl-histone-h3-lys18-antibody/9675
Histone H3, https://www.cellsignal.com/products/primary-antibodies/histone-h3-antibody/9715
SOD2/MnSOD (acetyl K68), https://www.abcam.com/sod2mnsod-acetyl-k68-antibody-epvanr2-ab137037.html
SOD2/MnSOD, https://www.abcam.com/sod2mnsod-antibody-ab13533.html
SIRT1, http://www.cloud-clone.com/products/PAE912Mu01.html
SIRT2, https://www.sigmaaldrich.com/DK/en/product/sigma/s8447
SIRT3, https://www.cellsignal.com/products/primary-antibodies/sirt3-d22a3-rabbit-mab/5490
SIRT4, http://www.cloud-clone.com/products/PAE914Hu01.html
SIRT5, https://www.cellsignal.com/products/primary-antibodies/sirt5-d8c3-rabbit-mab/8782
SIRT7, http://cloud-clone.com/products/PAE917Hu01.html
c-Jun, https://www.scbt.com/p/c-jun-antibody-h-79

GAPDH, https://www.scbt.com/p/gapdh-antibody-fl-335
Actin, https://www.sigmaaldrich.com/DK/en/product/sigma/a3854
Vinculin, https://www.cellsignal.com/products/primary-antibodies/vinculin-e1e9v-xp-rabbit-mab/13901
FoxO1, https://www.cellsignal.com/products/primary-antibodies/foxo1-c29h4-rabbit-mab/2880
FoxO3, https://www.cellsignal.com/products/primary-antibodies/foxo3a-d19a7-rabbit-mab/12829
Anti-Rabbit HRP, https://www.cellsignal.com/products/secondary-antibodies/anti-rabbit-igg-hrp-linked-antibody/7074
Anti-Mouse HRP, https://www.cellsignal.com/products/secondary-antibodies/anti-mouse-igg-hrp-linked-antibody/7076
Clean-Blot IP Detection Reagent, https://www.thermofisher.com/order/catalog/product/21230
anti-Rabbit IgG light chain HRP, https://www.cellsignal.com/products/secondary-antibodies/rabbit-anti-mouse-igg-light-chain-specific-d3v2a-mab-hrp-conjugate/58802
Myomesin, https://dshb.biology.uiowa.edu/mMaC-myomesin-B4
Atrogin-1, https://www.abcam.com/fbx32-antibody-ab74023.html
MuRF-1/2/3, https://www.abcam.com/murf1--murf3--murf2-antibody-epr64312-ab172479.html
Donkey anti-Mouse IgG (H+L), Alexa Fluor 488, Donkey anti-Mouse IgG (H+L), Alexa Fluor 488
Donkey anti-Rabbit IgG (H+L), DyLight 594, https://www.thermofisher.com/antibody/product/Donkey-anti-Rabbit-IgG-H-L-Cross-Adsorbed-Secondary-Antibody-Polyclonal/SA5-10040
Goat anti-Rabbit IgG (H+L), Alexa Fluor 546, https://www.thermofisher.com/antibody/product/Goat-anti-Rabbit-IgG-H-L-Highly-Cross-Adsorbed-Secondary-Antibody-Polyclonal/A-11035
Goat anti-Mouse IgG (H+ L), Alexa Fluor 488, https://www.thermofisher.com/antibody/product/Goat-anti-Mouse-IgG-H-L-Cross-Adsorbed-Secondary-Antibody-Polyclonal/A-11001
Myosin heavy chain Type I, https://dshb.biology.uiowa.edu/BA-D5
Myosin heavy chain Type IIA, https://dshb.biology.uiowa.edu/SC-71
Myosin heavy chain Type IIB, https://dshb.biology.uiowa.edu/BF-F3
Myosin heavy chain Type IIX IHC, https://dshb.biology.uiowa.edu/6H1
Goat anti-Mouse IgG1, Alexa Fluor 594, https://www.thermofisher.com/antibody/product/Goat-anti-Mouse-IgG1-Cross-Adsorbed-Secondary-Antibody-Polyclonal/A-21125
Goat anti-Mouse IgM (Heavy chain), Alexa Fluor 488, https://www.thermofisher.com/antibody/product/Goat-anti-Mouse-IgM-Heavy-chain-Cross-Adsorbed-Secondary-Antibody-Polyclonal/A-21042

Eukaryotic cell lines

Policy information about cell lines

Cell line source(s)	C2C12 (CRL-1722) and 293T (CRL-3216) were procured from ATCC.
Authentication	Commercial sources. Validation done by the supplier and we have not validated in our laboratory.
Mycoplasma contamination	All cell lines were tested negative for mycoplasma contamination.
Commonly misidentified lines (See ICLAC register)	No commonly misidentified cell line were used in the study.

Animals and other organisms

Policy information about studies involving animals; ARRIVE guidelines recommended for reporting animal research

Laboratory animals	C57BL/6, SIRT6fl/fl (Sirt6tm1.1Cxd/J) and ACTA1-Cre (FVB.Cg-Tg(ACTA1-care)79Jme/J) mice were procured from the Jackson Laboratories, USA. C57BL/6-SIRT6 mice was previously generated and C57BL/6 myogenin-cre mice was a gift from P. Puigserver.
Wild animals	Not used
Field-collected samples	Not used
Ethics oversight	Animal handling protocols were reviewed and duly approved by the Institutional Animal Ethics Committee (IAEC) of the Indian Institute of Science, constituted as per article number 13 of the Committee for the Purpose of Control and Supervision of Experiments on Animals (CPCSEA), Government of India. All the studies conformed with the Guide for the Care and Use of Laboratory Animals published by the US National Institute of Health (NIH Publication No. 85-23, revised 1996). The approved protocols are included in the manuscript.

Note that full information on the approval of the study protocol must also be provided in the manuscript.
