# Supplementary material for: Sirtuin 6 inhibition protects against glucocorticoid-induced skeletal muscle atrophy by regulating IGF/PI3K/AKT signaling
Source: Nat Commun. 2022 Sep 15;13:5415. doi: 10.1038/s41467-022-32905-w (PMC9478160; doi:10.1038/s41467-022-32905-w)
Supplement: Supplementary file 4 — Description of Additional Supplementary Files [file 41467_2022_32905_MOESM4_ESM.pdf]

Title: Supplementary Data 1:
Description: Statistical test details including ANOVA table analysis and p-value, and n value for each panel

Title: Supplementary Data 2:
Description: Details of primers, siRNAs, plasmids, adenovirus, kits, chemicals, reagents, equipments used in the study
